# Chemoreceptor family in plant-associated bacteria responds preferentially to the plant signal molecule glycerol 3-phosphate

**DOI:** 10.1186/s13059-025-03703-6

**Published:** 2025-08-29

**Authors:** Félix Velando, Jiawei Xing, Roberta Genova, Jean Paul Cerna-Vargas, Raquel Vázquez-Santiago, Miguel A. Matilla, Igor B. Zhulin, Tino Krell

**Affiliations:** 1 Department of Biotechnology and Environmental Protection, Estación Experimental del Zaidín, Consejo Superior de Investigaciones Científicas, Granada, Spain; 2 Department of Microbiology, The Ohio State University, Columbus, USA; 3 Translational Data Analytics Institute, The Ohio State University, Columbus, USA; 4 Centro de Biotecnología y Genómica de Plantas CBGP, Universidad Politécnica de Madrid-Instituto Nacional de Investigación y Tecnología Agraria y Alimentaria/CSIC, Parque Científico y Tecnológico de La UPM, Pozuelo de Alarcón, Madrid, Spain; 5 Simons Center for Quantitative Biology, Cold Spring Harbor Laboratory, Cold Spring Harbor, New York, NY 11724 USA

**Keywords:** Chemotaxis, Chemoreceptor, Plant-associated bacteria, Plant signal molecule, Glycerol 3-phosphate, Bioinformatics, Protein evolution, Isothermal titration calorimetry

## Abstract

**Background:**

Chemotaxis to plant compounds is frequently the initial step for the colonization of plants by bacteria. Plant pathogens and plant-associated bacteria contain approximately twice as many chemoreceptors as the bacterial average does, indicating that chemotaxis is particularly important for bacteria–plant interactions. However, information on the corresponding chemoreceptors and their chemoeffectors is limited.

**Results:**

We identify the chemoreceptor PacP from the phytopathogen *Pectobacterium atrosepticum*, which exclusively recognizes phosphorylated C3 compounds at its sCache ligand binding domain, mediating chemoattraction. Using a motif of PacP amino acid residues involved in ligand binding, we identify a chemoreceptor family, termed sCache_PC3, that is specific for phosphorylated C3 compounds. Isothermal titration calorimetry studies reveal that family members preferentially bind glycerol 3-phosphate, a key plant signaling molecule. Family members recognize glycerol 2-phosphate and glycolysis intermediates glyceraldehyde 3-phosphate, dihydroxyacetone phosphate, and 3-phosphoglycerate. This study presents the first evidence of chemoreceptors that bind phosphorylated compounds. We show that the sCache_PC3 family has evolved from an ancestral sCache domain that responds primarily to Krebs cycle intermediates. Members of the sCache_PC3 family are predominantly found in plant-associated bacteria, including many important phytopathogens belonging to the genera *Brenneria*, *Dickeya*, *Musicola*, *Pectobacterium*, and *Herbaspirillum*. Consistently, glycerol 3-phosphate is a signal molecule that is excreted by plants in response to stress and infection.

**Conclusions:**

Chemotaxis toward glycerol 3-phosphate may be a means for bacteria to localize stressed plants and move to infection sites. This study lays the groundwork for investigating the role of chemotaxis to phosphorylated C3 compounds in plant–bacteria interactions and virulence.

**Supplementary Information:**

The online version contains supplementary material available at 10.1186/s13059-025-03703-6.

## Background

Chemotaxis is the directed active movement of bacteria in response to chemical gradients. The primary benefit of chemotaxis is access to nutrients or the localization of sites that are favorable for growth [1]. However, chemotaxis has also been observed in response to signals such as quorum-sensing molecules, hormones, and neurotransmitters that provide the bacterium with useful information about its microenvironment [2–5]. Many bacteria establish interactions with other organisms, and chemotaxis to host compounds is frequently required for efficient host colonization or virulence [6, 7].

Chemotaxis is mediated by chemosensory pathways that contain as the central element the ternary complex comprising chemoreceptors, the CheA autokinase and the CheW coupling protein. Chemotactic signaling is typically initiated by signal binding at the ligand binding domain (LBD) of chemoreceptors that causes a molecular stimulus altering the autokinase activity of CheA and, consequently, the transphosphorylation of the response regulator CheY. In its phosphorylated state, CheY interacts with the flagellar motor, altering its activity and ultimately resulting in chemotaxis [8, 9].

The chemotactic sensory capacity of a bacterium is reflected in the number of chemoreceptors that can differ greatly among bacteria, ranging from 1 to 90 [10]. The bacterial lifestyle was found to determine the number of chemoreceptors [11, 12]. Whereas bacteria that inhabit specific ecological niches possess few chemoreceptors, bacteria that live in variable environments or that maintain interactions with other living species have many more chemoreceptors. Chemoreceptors respond to many structurally different compounds, including amino acids, organic acids, fatty acids, biogenic amines, polyamines, purine compounds, sugars, aromatic hydrocarbons, metal ions, inorganic anions, oxygen, or polysaccharides [13]. Although the link between chemoreceptor number and lifestyle has been established, we are still in the early stages of understanding the relationship between lifestyle and chemoreceptor function.

Chemotaxis is particularly important for the initiation of an interaction of beneficial [7] or pathogenic bacteria [6] with plants. The deletion of chemotactic signaling genes in different plant beneficial [14–17] and pathogenic bacteria [18–21] reduced bacterial plant colonization and infection. Frequently, chemotaxis is required for the efficient entry of bacteria into the plant host [22–24] and multiple lines of evidence indicate that compounds released by stomata and plant wounds attract bacteria to entry sites [6]. The importance of chemotaxis in plant infection is further supported by the fact that phytopathogens and plant-associated bacteria have very broad chemosensory capabilities [25]. On average, phytopathogens possess 27 chemoreceptors, twice as many as bacteria that are not associated with plants [25]. Many chemoreceptor families are almost exclusively present in phytopathogens, suggesting that they play specific roles in sensing plant compounds [25]. However, information on the signals recognized by chemoreceptors of plant-associated bacteria is scarce and this study aims to narrow this gap in knowledge.

We used *Pectobacterium atrosepticum* as a model phytopathogen. It is among the 10 most relevant plant pathogens, causing black leg and soft rot diseases [26]. The genome of *P. atrosepticum* strain SCRI1043 encodes 36 chemoreceptors that have a large variety of LBD types with different topologies [27]. To date, four chemoreceptors that respond to formate [28], quaternary amines [29], amino acids [30], and nitrate [31] have been identified. The functions of the remaining 32 chemoreceptors remain unknown.

Cache domains constitute the largest superfamily of extracytosolic LBDs in bacteria [32]. They assume either a monomodular (single Cache domain, sCache) or a bimodular configuration (double Cache domain, dCache). *P. atrosepticum* SCRI1043 has a single chemoreceptor with an sCache type LBD (ECA_RS12390). Several sCache LBDs from chemoreceptors have been studied in phylogenetically diverse bacterial species, where they recognize different C1 to C4 carboxylic acids [33–38], urea, and related compounds [39–41].

Over the last decade, the use of thermal shift assay-based ligand screening has become a very successful approach for identifying the functions of chemoreceptors [42–45]. In addition, we have recently pioneered a computational approach that aids in the identification of receptor signals. Using LBD/ligand 3D structural information and multiple alignments of homologous sequences, we derived sequence motifs that capture amino acid residues that interact with the ligand. Database searches for sequences that match these motifs have resulted in the identification of thousands of receptors that specifically bind amino acids [46], biogenic amines [47], purines [48], or formate [28]; these results were subsequently verified by isothermal titration calorimetry (ITC) studies.

In the present study, we combined both approaches. Thermal shift assays and ITC studies of the LBD of ECA_RS12390 (termed PacP) revealed that it specifically binds phosphorylated C3 compounds, including three glycolysis intermediates as well as glycerol 2-phosphate and glycerol 3-phosphate. Using a specific sequence motif present in the binding site, we defined the corresponding domain family and confirmed the ligand binding characteristics of the selected members. Family members are almost exclusively present in plant-associated bacteria, including a number of important plant pathogens.

Glycerol 3-phosphate was preferentially recognized by the analyzed family members and induced the strongest chemotaxis in *P. atrosepticum*. Glycerol 3-phosphate is one of the most relevant plant signaling molecules. For example, it regulates systemic immunity [49, 50] and responses to drought [51, 52]. To the best of our knowledge, this is the first report on a bacterial receptor family that specifically binds phosphorylated compounds.

## Results

### The chemoreceptor ECA_RS12390 (PacP) exclusively binds phosphorylated C3 compounds

**Fig. 1:**
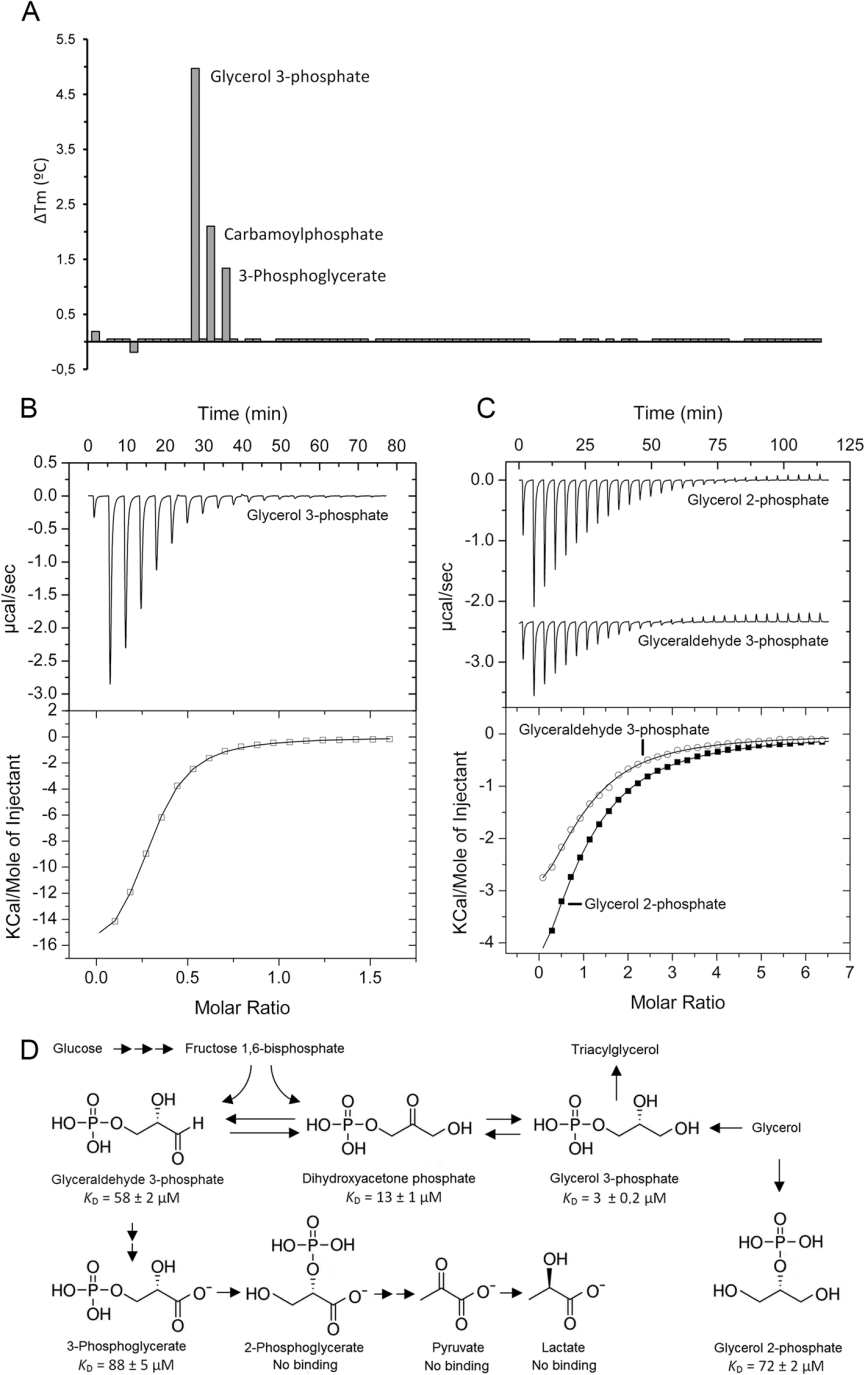
Binding of phosphorylated C3 compounds to the ligand binding domain of the chemoreceptor ECA_RS12390 (PacP-LBD). **A** Thermal shift assays with compounds of the Biolog array PM4A (phosphorus and sulfur sources). Tm changes with respect to the ligand-free protein are shown. **B**, **C** Microcalorimetric titration of 80 μM ECA_RS12390-LBD with 8 µL aliquots of 1 mM glycerol 3-phosphate, 2 mM glycerol 2-phosphate, and 2.5 mM glyceraldehyde 3-phosphate. Upper panels: Titration raw data. Lower panels: Concentration-normalized and dilution heat-corrected integrated titration data. The lines are the best fits with the “One binding site model” of the MicroCal version of ORIGIN. **D** Summary of ligands recognized by PacP-LBD and their metabolic relationships

Chemoreceptor ECA_RS12390 is composed of a periplasmic sCache_2 LBD (PF17200), flanked by two transmembrane regions, and a cytosolic signaling domain (PF00015) at is C-terminal extension. To identify the ligands recognized by this chemoreceptor, its individual LBD was overexpressed in *Escherichia coli* and purified by affinity chromatography. The purified LBD was subsequently subjected to thermal shift-based ligand screening assays using the compound arrays PM1, PM2A, PM3B, and PM4A, which contain bacterial carbon, nitrogen, phosphorus, and sulfur sources (Additional file 1: Fig. S1). This approach measures ligand-induced increases in protein thermal stability, as quantified by the midpoint of the thermal unfolding transition (Tm). Among the 95 phosphorylated or sulfonated compounds in the PM4A compound array, glycerol 3-phosphate, carbamoylphosphate, and 3-phosphoglycerate caused significant increases in Tm (Fig. 1A). No significant increases in Tm were observed for the remaining compound arrays, which include most of the carboxylic acids that were previously shown to bind to sCache_2 domains [33–38].

We subsequently conducted ITC binding studies to derive the dissociation constant (*K*_D_). The titration of ECA_RS12390-LBD with glycerol 3-phosphate resulted in large exothermic heat changes (Fig. 1B), leading to a calculated *K*_D_ of 3 ± 0.2 µM (Table 1). The binding of 3-phosphoglycerate occurred with significantly lower affinity (*K*_D_ = 88 ± 5 µM), whereas titration with carbamoylphosphate did not result in measurable heat release. Owing to the restriction of ligand dilution heats, microcalorimetry only permits monitoring of high-affinity binding events, indicating that carbamoylphosphate may bind with an affinity that is not detectable by ITC.

**Table 1:** Dissociation constants (*K*_D_) derived from microcalorimetric binding studies of phosphorylated compounds to the LBDs of different chemoreceptors

Rec. no	Accession code	Species	*K*_D_ (µM)				
			Glyceraldehyde 3-P	Dihydroxyacetone P	3-P glycerate	Glycerol 3-P	Glycerol 2-P
R1	WP_011094075.1 (PacP, ECA_RS12390)	*P. atrosepticum*	58 ± 2	13 ± 1	88 ± 5	3 ± 0.2	72 ± 2
R2	WP_136157342.1	*Brenneria roseae*	253 ± 11	99 ± 10	261 ± 17	11 ± 0.2	90 ± 3
R3	WP_006464688.1	*Herbaspirillum frisingense*	Nb	109 ± 14	Nb	33 ± 1	36 ± 2
R4	WP_158281851.1	*Rivicola pingtungensis*	100 ± 2	230 ± 10	926 ± 8	7 ± 0.2	60 ± 2
R5	WP_028444678.1	*Chitinimonas koreensis*	Nb	Nb	Nb	Nb	Nb
R6	WP_174062248.1	*Agrobacterium larrymoorei*	Nb	Nb	Nb	Nb	Nb
R7	WP_019915375.1	*Methyloversatilis discipulorum*	126 ± 9	Nb	Nb	Nb	223 ± 45
R8	WP_028536266.1	*Paludibacterium yongneupense*	No protein overexpression				
R9	WP_189529961.1	*Paludibacterium paludis*	Nb	Nb	Nb	Nb	Nb
R10	WP_028866120.1	*Psychromonas aquimarina*	Nb	Nb	Nb	Nb	Nb

*Nb* no binding

We next explored the binding of structurally related phosphorylated compounds that were not present in the compound arrays. No binding was observed for 2-phosphoglycerate, whereas glycerol 2-phosphate and glyceraldehyde 3-phosphate bound with *K*_D_ values of 72 ± 2 and 58 ± 2 µM, respectively (Fig. 1C, Table 1). In addition, dihydroxyacetone phosphate was recognized with a *K*_D_ of 13 ± 1 µM. Pyruvate and lactate, ligands recognized by other sCache LBDs [34–36, 53], did not bind, suggesting that a phospho-moiety is required for binding. In summary, ECA_RS12390 binds specifically to phosphorylated C3 compounds (Fig. 1D) and, to our knowledge, represents the first chemoreceptor that binds exclusively to phosphorylated ligands.

Glyceraldehyde 3-phosphate, dihydroxyacetone phosphate, and 3-phosphoglycerate are glycolysis intermediates (Fig. 1D). Glycerol 3-phosphate is an intermediate that connects glycolysis, glycerol metabolism, and triacylglycerol synthesis [54, 55]. Furthermore, glyceraldehyde 3-phosphate and 3-phosphoglycerate are intermediates of the Calvin–Benson–Bassham cycle that permits CO_2_ fixation in plants [56]. We renamed ECA_RS12390 PacP (*Pectobacterium atrosepticum *chemoreceptor for phosphorylated compounds).

### PacP mediates chemoattraction to phosphorylated compounds that bind to its LBD

**Fig. 2:**
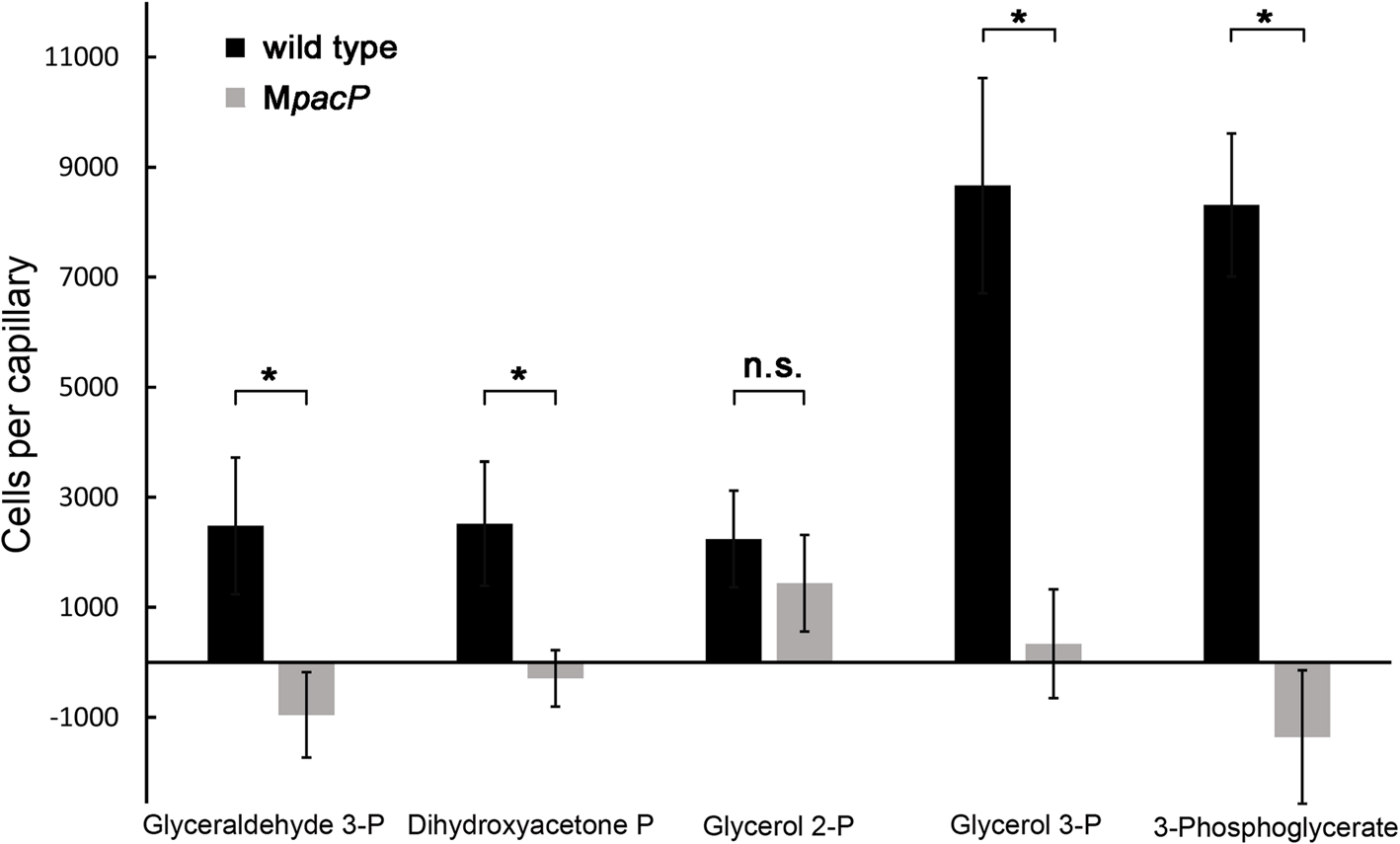
Capillary chemotaxis assays of *P. atrosepticum* SCRI1043 and a mutant deficient in *pacP* to 1 mM solutions of the identified ligands. The data have been corrected with the number of cells that swam into buffer-containing capillaries. The data are presented as the means and standard deviations from three biological replicates conducted in triplicate (*P value < 0.01; by Student’s *t*-test, n.s.: not significant)

We conducted quantitative capillary chemotaxis assays to determine whether PacP ligands induce chemotaxis. Modest chemoattraction was observed for glyceraldehyde 3-phosphate, dihydroxyacetone phosphate, and glycerol 2-phosphate. In contrast, chemotaxis toward glycerol 3-phosphate and 3-phosphoglycerate was significantly greater (Fig. 2). To assess the contribution of PacP to this response, we constructed a *pacP* mutant (M*pacP*). Only glycerol 2-phosphate served as an attractant for the mutant, indicating that PacP is the sole chemoreceptor for glyceraldehyde 3-phosphate, dihydroxyacetone phosphate, glycerol 3-phosphate, and 3-phosphoglycerate (Fig. 2). An additional chemoreceptor(s) must exist for glycerol 2-phosphate. These responses are mediated by the sole chemosensory pathway present in the strain SCRI1043, since a mutant in *cheA* failed to respond to glycerol 3-phosphate and 3-phosphoglycerate (Additional file 1: Fig. S2). In trans complementation of M*pacP* with a plasmid harboring the *pacP* gene restored chemotaxis to wild type levels (Additional file 1: Fig. S2).

### Three PacP ligands are of metabolic value

**Fig. 3:**
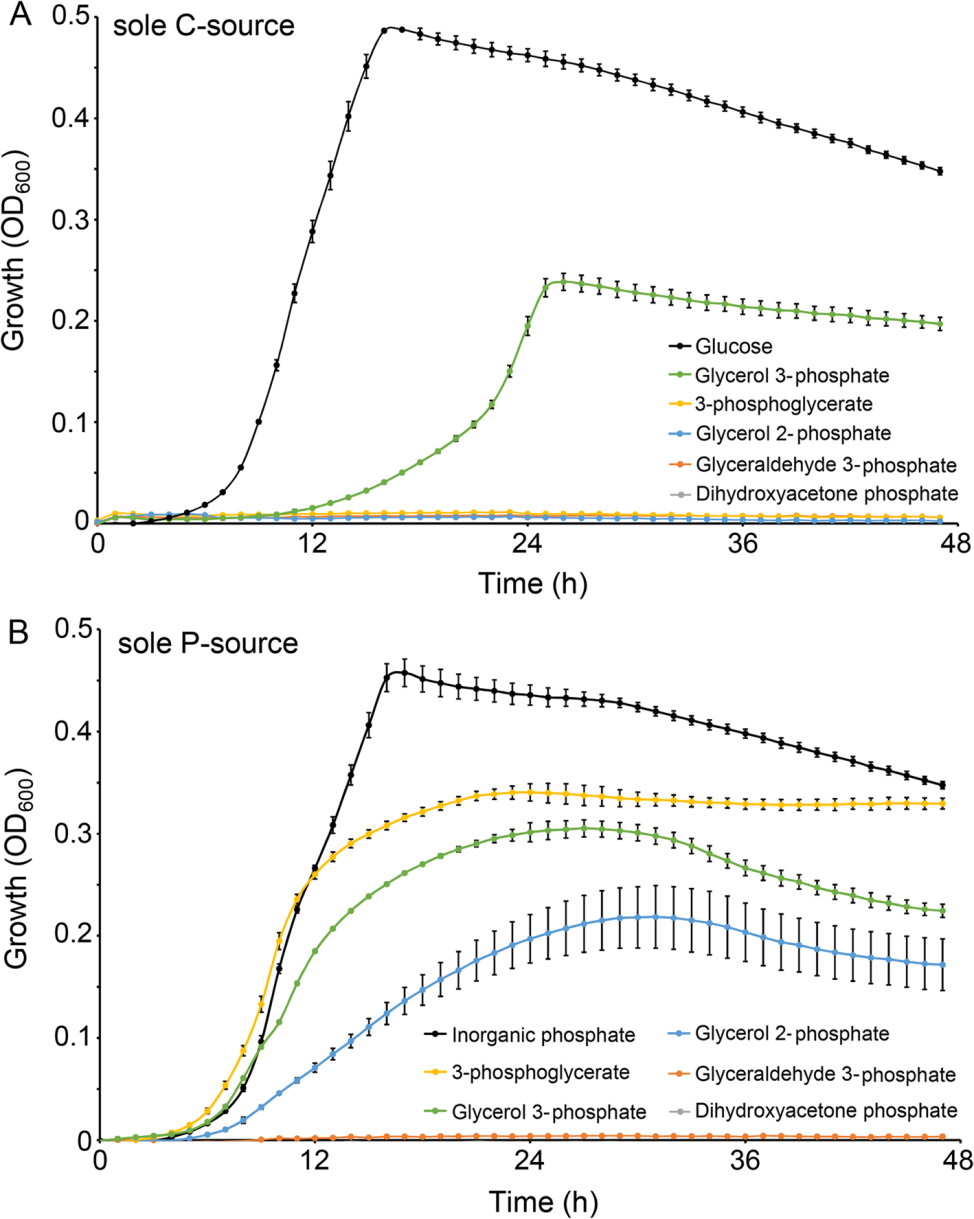
Growth of *Pectobacterium atrosepticum* SCRI1043 when the PacP ligands were used as the sole carbon (**A**) or phosphorus (**B**) source. As a reference, growth was monitored on glucose and inorganic phosphate as the sole C or P source, respectively. The data are the means and standard deviations of three biological replicates

Chemoeffectors can be classified into three groups: (i) compounds that are of metabolic value, (ii) compounds that act as environmental signaling cues, and (iii) compounds with a dual metabolic/signal function [1, 57]. To assess the metabolic value of the PacP ligands, we conducted growth experiments in minimal medium containing the individual ligands as the sole carbon or phosphorus source. Glycerol 3-phosphate was the only ligand that supported growth as the sole carbon and phosphorus source, whereas 3-phosphoglycerate and glycerol 2-phosphate permitted growth as phosphorus sources (Fig. 3). The glycolysis intermediates glyceraldehyde 3-phosphate and dihydroxyacetone phosphate were devoid of apparent metabolic value (Fig. 3).

### Definition of the sCache_PC3 family for phosphorylated compounds

Subsequent experiments were conducted to define the LBD family that binds phosphorylated C3 compounds. LBDs are rapidly evolving domains, and their ligand specificity is thus poorly reflected in overall sequence identity [58]. We have recently established a procedure to predict ligands recognized by LBDs by taking into account the amino acid residues in the binding site that interact with the bound ligand [28, 46–48]. Because extensive attempts to crystallize PacP-LBD failed, we constructed an AlphaFold2 model [59], which was then used for computational ligand docking.

**Fig. 4:**
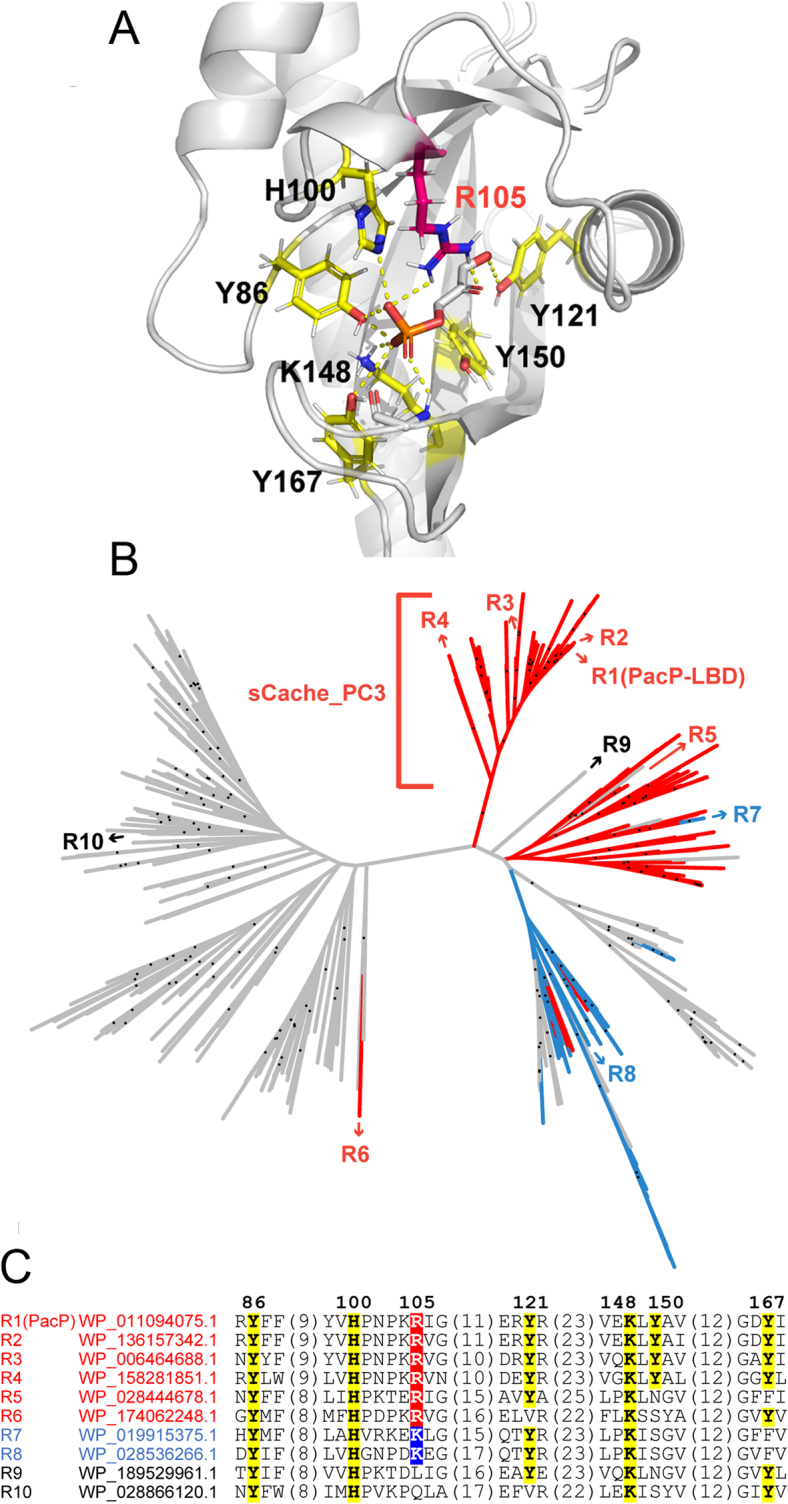
Definition of the sCache_PC3 family.** A** PacP-LBD AlphaFold2 model containing docked dihydroxyacetone phosphate. Key residues are labeled.** B** Maximum likelihood tree of sCache_2 domains from PacP homologs. Receptors R1 to R10, which were selected for experimental studies, are labeled. Dots indicate branches with bootstrap values not less than 70. **C** Multiple sequence alignment of the sequence fragments covering the binding pocket of R1 to R10. The color of the protein name corresponds to that of **B**: red: conserved Arg105; blue: Lys instead of Arg105; gray: no Arg or Lys at position Arg105. PacP residue numbering is shown

These experiments indicated that Arg105 in PacP is likely the key residue for phosphate sensing (Fig. 4A). A number of other amino acid residues, Y86, H100, Y121, K148, Y150, and Y167, also interact with the bound ligand (Fig. 4A). To identify other chemoreceptors that may bind phosphorylated compounds, we collected > 1000 PacP homologs from the RefSeq protein database, from which 610 nonredundant sCache_2 domains were used to construct a phylogenetic tree (Fig. 4B). To examine which of these domains might recognize phosphorylated compounds similarly to PacP-LBD, we selected 10 domains from chemoreceptors from different clades (R1 to R10, Fig. 4B, C, Table 1) for further analyses. The corresponding source strains were α-, β-, and γ-proteobacteria that were isolated from plants, soil, or freshwater (Additional file 2: Table S1) [60–73]. Domains were selected for their pattern of conservation of residues within the predicted binding site (Fig. 4A) from the three distinct groups that are shown in different colors in Fig. 4B, C. Domains shown in red (including R1 to R6) have an invariant Arg105 (Fig. 4B, C). Domains shown in blue (including R7 and R8) have an Arg105Lys substitution (Fig. 4B, C). Domains shown in gray (including R9 and R10) have various other substitutions at the PacP position Arg105 (Fig. 4B, C).

**Fig. 5:**
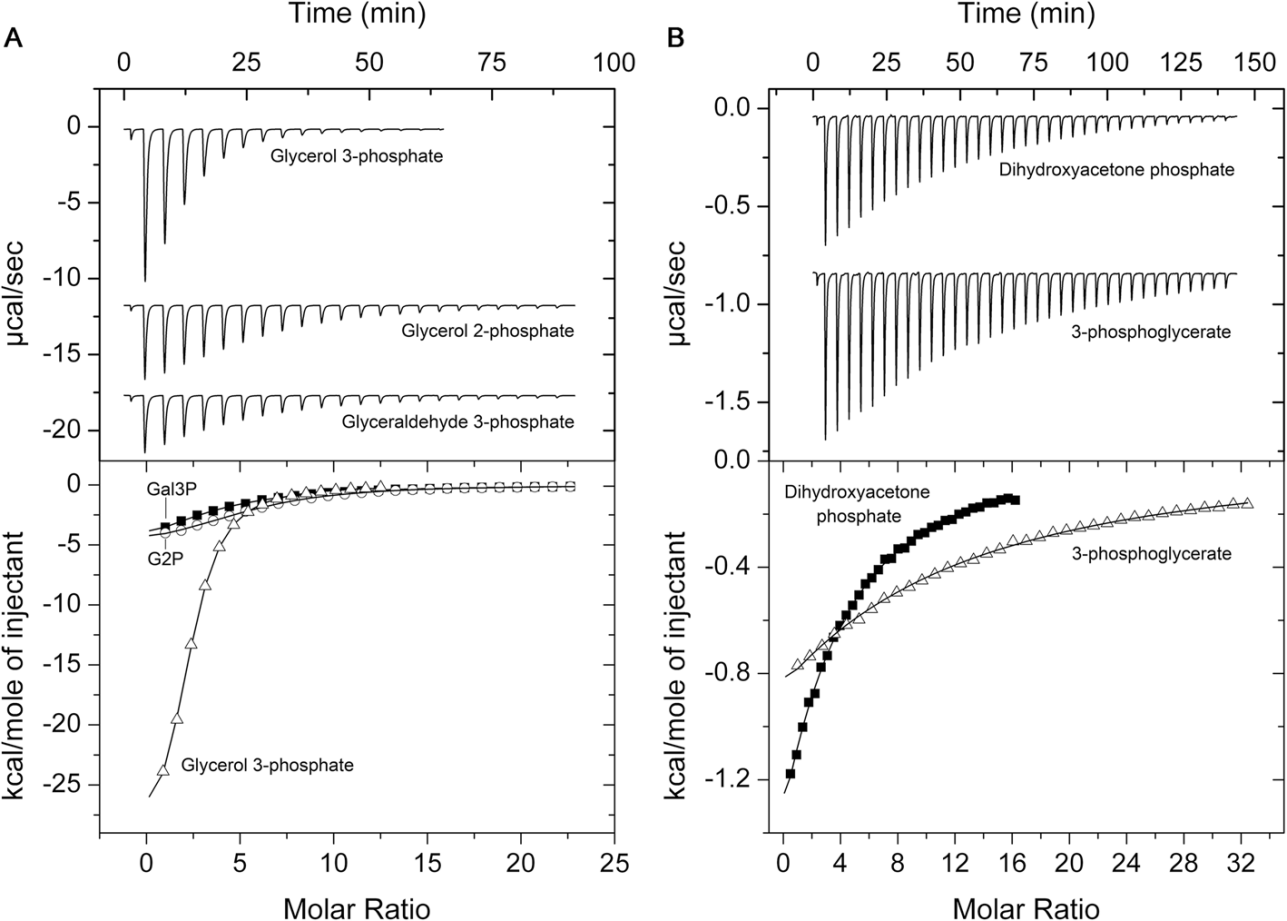
Microcalorimetric titration of the ligand binding domain of R4 with different phosphorylated compounds. **A** Titration of 33 µM protein with 8 µL aliquots of 3 mM glycerol 3-phosphate, 5 mM glycerol 2-phosphate (G2P), and 5 mM glyceraldehyde 3-phosphate (Gal3P). **B** Titration of 33 µM protein with 8 µL aliquots of 2.5 mM dihydroxyacetone phosphate and 5 mM 3-phosphoglycerate. Upper panels: Titration raw data. Lower panels: Concentration-normalized and dilution heat-corrected integrated titration data. The lines are the best fits with the “One binding site model” of the MicroCal version of ORIGIN

To identify the ligands recognized by R2 to R10, expression plasmids harboring codon-optimized LBD sequences were introduced into *E. coli*. With the exception of R8, all the proteins could be overexpressed. The purified proteins were then subjected to microcalorimetric titrations. Receptors R2, R3, and R4 all bound phosphorylated compounds. Whereas R2 and R4 bound all five PacP ligands, receptor R3 bound only three of them: glycerol 3-phosphate, glycerol 2-phosphate, and dihydroxyacetone phosphate (Table 1). PacP, R2, R3, and R4 recognized glycerol 3-phosphate with the highest affinity, with dissociation constants ranging from 3 to 33 µM. These affinities are in the range typically observed for sensor protein–ligand interactions [74]. Glycerol 2-phosphate was the second most tightly binding ligand for the three proteins. The preferential recognition of glycerol 3-phosphate is illustrated by the binding studies of the 5 phosphorylated ligands to R4 (Fig. 5).

To determine whether receptors R2, R3, and R4 recognize other ligands in addition to the phosphorylated compounds, we conducted thermal shift assays with the compound arrays PM1, PM2A, PM3B, and PM4A (Additional file 1: Fig. S1). Glycerol 3-phosphate was the only compound that caused significant increases in the Tm for proteins R3 and R4 (Additional file 1: Fig. S3). In addition to glycerol 3-phosphate, several small acids caused minor increases in the Tm of R2 (Additional file 1: Fig. S3). However, ITC studies of these compounds to R2 showed binding exclusively for glycerol 3-phosphate. Taken together, the data show that proteins R2, R3, and R4, like PacP, recognize exclusively phosphorylated C3 compounds.

Among the remaining proteins, only R7 bound to phosphorylated compounds, namely, glyceraldehyde 2-phosphate and glycerol 3-phosphate. However, the affinities were well below the affinities of the tightest binding ligands of receptors R1 to R4 (Table 1). These data indicate that the complete sequence motif present in R1 to R4 (Fig. 4C) is a prerequisite for high-affinity binding of phosphorylated compounds. To verify the role of this sequence motif in ligand binding, we generated a PacP-LBD mutant in which Y86, H100, R105, Y121, K148, and Y167 had been replaced by alanine residues. Microcalorimetric titrations of this domain with glycerol 3-phosphate and glyceraldehyde 3-phosphate showed heat changes that are indistinguishable from ligand dilution heats, indicating an absence of binding (Additional file 1: Fig. S4). The corresponding domain family has been termed sCache_PC3 (sCache domains for phosphorylated C3 compounds). The members of this family are provided in Additional file 3 and are exclusively found in chemoreceptors.

### The sCache_PC3 family has arisen from domains that bind different carboxylic acids

**Fig. 6:**
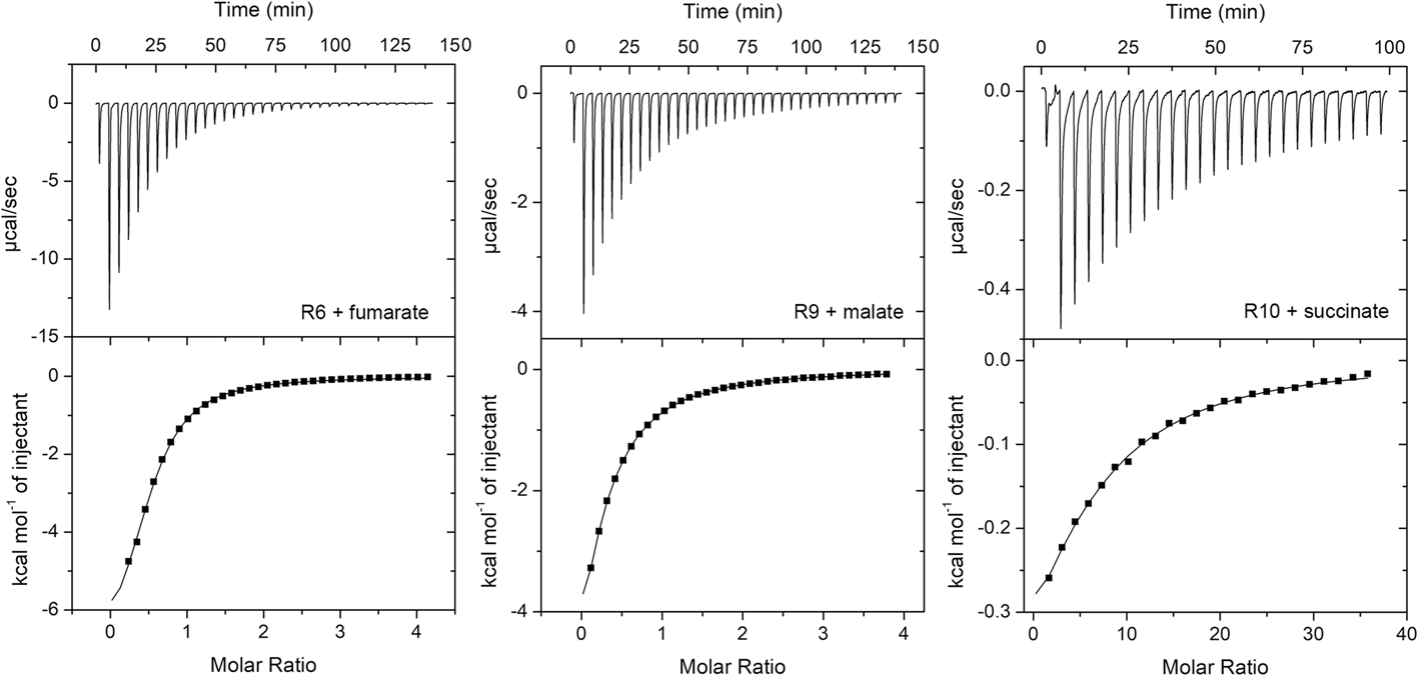
Microcalorimetric titrations of the ligand binding domain of different receptors that do not recognize phosphorylated compounds with Krebs cycle intermediates. Upper panels: Titration of 42 to 517 μM protein with 8 μL aliquots of 5 to 10 mM ligand solutions. The proteins and ligands are indicated. Lower panels: Concentration-normalized and dilution heat-corrected titration data. The dissociation constants are provided in Table 2

Because R5, R6, R9, and R10 fail to bind phosphorylated compounds, efforts have been made to identify their ligands. To achieve this goal, the purified proteins were analyzed by a thermal shift assay using the compound arrays PM1, PM2, PM3B, and PM4. The compounds that caused significant Tm shifts were selected for microcalorimetric studies. All four receptors bound one or several Krebs cycle intermediates, such as succinate, fumarate, malate, or citrate (Fig. 6, Table 2). In addition, three domains bound other organic acids (Table 2); three of which, tricarballylate, methyl-, and bromosuccinate, are structurally related to Krebs cycle intermediates. The affinities of these compounds were generally lower than those of Krebs cycle intermediates (Table 2). Taken together, these findings suggest that domains other than those of the sCache_PC3 family (Fig. 4B) respond to Krebs cycle intermediates. The sCache_PC3 family likely evolved from these receptors, which are found in Burkholderiales and Enterobacterales.

**Table 2:** Dissociation constants (*K*_D_) derived from microcalorimetric binding studies of organic acids to the LBDs of different chemoreceptors

Rec. no	*K*_D_ (µM)								
	Succinate	Fumarate	Malate	Citrate	Tricarballylate	Bromosuccinate	Methylsuccinate	Glycolate	L-lactate
R5	127 ± 5	68 ± 6	61 ± 2	10 ± 2	15 ± 1	Nb	Nb	Nb	Nb
R6	90 ± 4	90 ± 4	63 ± 1	Nb	Nb	Nb	Nb	Nb	Nb
R9	Nb	Nb	185 ± 5	Nb	138 ± 33	200 ± 13	254 ± 53	Nb	Nb
R10	362 ± 23	Nb	Nb	Nb	Nb	Nb	Nb	322 ± 114	463 ± 51

*Nb* no binding

### sCache_PC3 members are present primarily in plant-associated bacteria

**Fig. 7:**
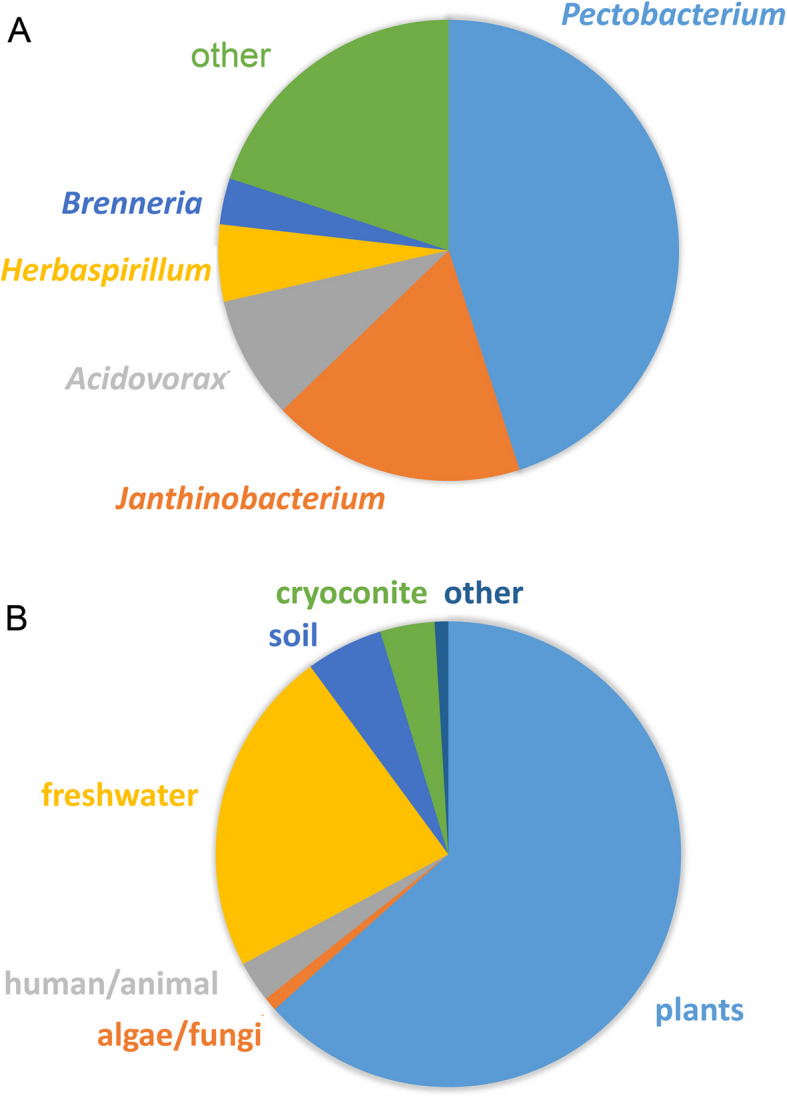
sCache_PC3 family of sensor domains. **A** Distribution of family members in different bacterial genera. Genera with fewer than 10 members are combined as “other.” **B** Isolation sources of strains harboring chemoreceptors with sCache_PC3 domains. Detailed information is provided in Additional file 3

Members of the sCache_PC3 family are chemoreceptors from γ-proteobacteria that belong to the orders Burkholderiales and Enterobacterales (Additional file 3). At the genus level, the most abundant bacteria were *Pectobacterium*, followed by *Janthinobacterium*, *Acidovorax*, *Herbaspirillum*, and *Brenneria* (Fig. 7A). To gain insight into the environments inhabited by these bacteria, we compiled the isolation sources of all the family members (Additional file 3, Fig. 7B). Importantly, approximately two-thirds of the strains were isolated from plants, and another one quarter were isolated from freshwater and soil, which are two habitats in which plants are frequently found. In a number of cases, strains of the same species were isolated from plants and soil or freshwater [75, 76]. Among the plant-associated strains, many belong to species that are associated with plant virulence, such as *Brenneria*, *Dickeya*, *Musicola*, *Pectobacterium*, *Herbaspirillum*, *Acidovorax*, and *Paracidovorax* [77, 78] (Additional file 3). Despite the presence of many human and animal derived strains in databases, only very few of them possess receptors with an sCache_PC3 domain (Fig. 7B), supporting the notion that sCache_PC3-containing chemoreceptors are predominantly present in plant-associated bacteria.

## Discussion

The functions of flagellar motility and chemotaxis in bacteria are very diverse and depend on bacterial lifestyles [57]. Accessing nutrients and, more generally, locating niches that are optimal for growth appear to be the major functions of bacterial chemotaxis. Chemotaxis is also required for interdomain communication, permitting the establishment of beneficial or pathogenic interactions of bacteria with humans, animals, and plants [6, 57]. The diversity of functions of chemotaxis is reflected by the observation that bacteria of different lifestyles differ largely in the number and type of chemoreceptors [11, 12]. Whereas bacteria that inhabit a specific ecological niche possess few chemoreceptors, bacteria that live in variable environments or that maintain interactions with other living species have many more chemoreceptors. However, since the functions of most chemoreceptors are unknown, our understanding of the link between chemoreceptor function and lifestyle is currently limited.

Plant pathogens and plant-associated bacteria stand out for their chemosensory capacities because they possess approximately twice as many chemoreceptors as the bacterial average [25]. Many chemoreceptor families have been found to be present primarily in plant-associated bacteria or phytopathogens [25]. However, the ligands they recognize remain largely unknown. The identification of a chemoreceptor family in plant-associated bacteria that responds to the plant signal glycerol 3-phosphate provides novel insight. In plants, glycerol 3-phosphate is a critical inducer of systemic acquired resistance (SAR) to pathogen attack; mutants defective in glycerol 3-phosphate synthesis are unable to induce SAR [49, 50, 79]. Plant infection induces SAR by stimulating glycerol 3-phosphate biosynthesis via the upregulation of its biosynthetic genes [80]. Glycerol 3-phosphate is also abundant in the rhizosphere, and it is the only PacP ligand that can be detected in root exudates [81, 82].

What might be the physiological benefit of chemotaxis to glycerol 3-phosphate for plant pathogens? (i) Our studies show that *P. atrosepticum* can use glycerol 3-phosphate as the sole carbon and phosphorus source for growth and that access to nutrients may be beneficial. (ii) Glycerol 3-phosphate, which accumulates at the site of infection [80], likely attracts bacteria to these locations, facilitating plant entry. (iii) Glycerol 3-phosphate is a stress-related signaling molecule, and its levels increase in response to phosphate starvation [81] and drought [51]. Among the 114 compounds enriched in the drought-treated endosphere, glycerol 3-phosphate was increased approximately 22,000-fold. It was thus the compound with by far the most significant increase. Further experiments revealed that this increase was due to changes in the host and not within the rhizosphere [51]. Because it is a stress signal, chemotaxis to glycerol 3-phosphate may also be a means to locate and move to stressed plants, which are easier targets for infection than non-stressed plants. Glycerol 3-phosphate is also an important bacterial signaling molecule. For example, in *P. aeruginosa*, glycerol 3-phosphate accumulation affects twitching motility, pyocyanine and exopolysaccharide biosynthesis, antibiotic resistance, and tolerance to oxidative stress [83]. Further studies will explore the degree to which glycerol 3-phosphate impacts the physiology of plant-associated bacteria.

The lack of information on signals that stimulate bacterial receptors is currently a major limitation in microbiology and represents an important research need. Here, we combined two experimental strategies that were successfully used in the past to identify receptor signals, namely, thermal shift-based high-throughput ligand screening [42, 44, 45] and the use of binding pocket amino acid motifs derived from LBD signal 3D structures [28, 46–48]. In contrast to the latter three studies, which were based on experimental three-dimensional LBD signal co-structures, we identified a sequence motif in a ligand-docked AlpaFold2 model of PacP-LBD. This approach thus does not require experimental protein structures, suggesting that it might be universally applicable for identifying receptor signals. The sCache_PC3 family is characterized by the Y(13)H(4)R(15)Y(26)K(1)Y(16) sequence motif (Fig. 4C), which can be used to identify additional family members. Combining these two strategies is thus a very potent approach for identifying novel LBD families, providing insight into receptor function. This approach is not restricted to chemoreceptors and can be used to identify signals for LBDs of any other receptor family.

Chemotaxis has thus far been observed for many compound families, including amino acids, organic acids, fatty acids, sugars, polyamines, quaternary amines, purines, pyrimidines, aromatic hydrocarbons, oxygen, inorganic ions, and polysaccharides [13, 84]. This is the first report of chemoreceptors that recognize specifically phosphorylated compounds, expanding our knowledge of the sensory capacity of chemoreceptors. Previous studies reported weak chemotaxis of *Bdellovibrio bacteriovorus* and *Bacillus subtilis* to 3-phosphoglycerate, glycerol 3-phosphate, and glycerol 2-phosphate [85, 86]. However, these bacteria do not possess sCache_PC3-containing chemoreceptors (Additional file 3), indicating that there are other types of chemoreceptors that mediate such responses. To the best of our knowledge, this is the first report of chemotaxis to the glycolysis intermediates glyceraldehyde 3-phosphate and dihydroxyacetone phosphate.

The identification of the sCache_PC3 family is a significant contribution to the field of plant–microbe interactions and provides the basis for further studies exploring the physiological relevance of bacterial chemotaxis to phosphorylated C3 compounds. It will also serve as an experimental guide to define other domain families.

## Conclusions

The bacterial lifestyle has shaped the evolution of signal transduction systems, and the number and type of chemoreceptors varies greatly between bacteria occupying various ecological niches. Our understanding of the relationship between lifestyle and chemoreceptor function is limited. Chemotaxis is particularly important for the initial stages of plant–bacteria interaction. Our discovery of a chemoreceptor family in plant-associated bacteria that primarily responds to an important plant signal molecule is a significant advancement. This study allows for further investigations to determine the physiological relevance of the observed chemotaxis for plant–bacteria interaction. The lack of knowledge about signals recognized by bacterial receptors is currently a major challenge in microbiology. This study illustrates the potential of combining experimental ligand screening with computational ligand prediction to identify signals recognized by uncharacterized receptors.

## Methods

### Bacterial strains and growth conditions

The bacterial strains used in this study are listed in Additional file 2: Table S2 [87–92]. *P. atrosepticum* SCRI1043 and its derivative strains were grown at 30 °C in Luria broth (5 g/L yeast extract, 10 g/L bacto tryptone, and 5 g/L NaCl) or minimal medium (0.41 mM MgSO_4_, 7.56 mM (NH_4_)_2_SO_4_, 40 mM K_2_HPO_4_, and 15 mM KH_2_PO_4_) supplemented with 0.2% (w/v) glucose as a carbon source. *E. coli* strains were grown at 37 °C in LB. When appropriate, antibiotics were used at the following final concentrations (in μg/mL): kanamycin, 50; ampicillin, 100; and streptomycin, 50.

### Plasmids and mutants

A mutant defective in *ECA_RS12390* was constructed by homologous recombination using a derivative plasmid of the suicide vector pKNG101. Briefly, a 0.5-kb fragment corresponding to the region encoding the LBD of ECA_RS12390 was amplified by the primers specified in Additional file 2: Table S2 and cloned into pGEM®-T. The resulting plasmid pGEM::ECA_RS12390 was digested with XmaI, and the 0.5-kb insert was cloned into the same site at pKNG101 to generate pKNG101::ECA_RS12390. This plasmid was subsequently transformed into *P. atrosepticum* SRCI1043 via biparental conjugation using *E. coli* β2163. All the plasmids were verified by PCR and sequencing. The pETb(+) derivatives encoding the LBD of the different receptors analyzed in this study (Table 1) were purchased from GeneScript. Plasmid pBBR-ECA_RS12390 was constructed by amplifying the *pacP* gene using the primers detailed in Additional file 2: Table S2 and cloning of the PCR product into the NdeI/BamHI sites of pBBR1MCS2_START. The sequences of the proteins analyzed in this study are provided in Additional file 2: Table S3. The ECA_RS12390 nucleotide sequence is available in the published genome of *P. atrosepticum* SCRI1043 [60]*.*

### Protein overexpression and purification

Plasmids for the overexpression of the different proteins were transformed into *E. coli* BL21(DE3). The resulting strains were grown under continuous stirring (200 rpm) at 30 °C in 2-L Erlenmeyer flasks containing 500 mL of LB medium supplemented with 50 μg/mL kanamycin. At an OD_660_ of 0.5, protein expression was induced by the addition of 0.1 mM isopropyl β-D-1-thiogalactopyranoside. Growth was continued at 16 °C overnight prior to cell harvesting by centrifugation at 10,000 × *g* for 20 min. Cell pellets were resuspended in buffer A (Additional file 2: Table S4) and subsequently broken by French press treatment at 62.5 lb/in^2^. After centrifugation at 20,000 × *g* for 30 min, the supernatants were loaded onto 5-mL HisTrap HP columns (Amersham Biosciences) equilibrated with buffer A, and the proteins were eluted with a linear gradient of buffer B (Additional file 2: Table S4). All proteins contain a hexa-histidine tag at the N-terminal extension (Additional file 2: Table S3) and were purified at 4 °C. The purified proteins were dialyzed overnight into the corresponding analysis buffers (Additional file 2: Table S4) for immediate analysis.

### Differential scanning fluorimetry-based thermal shift assays

Assays were carried out on a MyIQ2 Real-Time PCR instrument (Bio-Rad, Hercules, CA, USA). Ligand solutions were prepared by dissolving the array compounds in 50 µL of Milli-Q water, which, according to the manufacturer, corresponds to a concentration of 10–20 mM. Freshly purified proteins were dialyzed into analysis buffer (Additional file 2: Table S4). Compound arrays PM1, PM2A (carbon sources), PM3B (nitrogen sources), and PM4A (phosphorus and sulfur sources) from Biolog (https://www.biolog.com/) were used. The compositions of these arrays are provided in Additional file 2: Table S1. The experiments were conducted in 96-well plates, and each assay mixture contained 20 µL of the dialyzed protein (at 80–50 µM), 2 µL of 5 × SYPRO orange (Life Technologies, Eugene, Oregon, USA), and 2.5 µL of the resuspended array compound or the equivalent amount of buffer in the ligand-free control. The samples were heated from 23 to 85 °C at a scan rate of 1 °C/min. The protein unfolding curves were monitored by detecting changes in SYPRO® Orange fluorescence. The Tm values were determined from the first derivative values of the raw fluorescence data.

### Isothermal titration calorimetry (ITC)

All experiments were conducted on a VP microcalorimeter (Microcal, MA) at 15 °C for PacP-LBD and at 20 °C for the remaining proteins. Proteins were dialyzed into the analysis buffers specified in Additional file 2: Table S4, placed into the sample cell and titrated with aliquots of ligand solutions (1 to 10 mM) freshly prepared in dialysis buffer. The mean enthalpies measured from the injection of the ligands into the analysis buffer were subtracted from the raw titration data prior to data analysis with the MicroCal version of ORIGIN. The data were fitted with the “One binding site model” of ORIGIN.

### Quantitative capillarity chemotaxis assays

Overnight cultures of *P. atrosepticum* SCRI1043 were grown at 30 °C in minimal medium. At an OD_660_ of 0.4–0.45, the cultures were washed twice with chemotaxis buffer (50 mM K_2_HPO_4_/KH_2_PO_4_, 20 μM EDTA, 0.05% (v/v) glycerol, pH 7.0) and diluted to an OD_660_ of 0.1 in the same buffer. Subsequently, 230 μL of the resulting bacterial suspension was placed into the wells of 96-well plates. One-microliter capillary tubes (P1424, Microcaps; Drummond Scientific) were heat-sealed at one end and filled with either chemotaxis buffer (negative control) or chemotaxis buffer containing PacP ligands at a concentration of 1 mM. The capillaries were immersed in the bacterial suspensions at their open ends. After 30 min at room temperature, the capillaries were removed from the bacterial suspensions, rinsed with sterile water, and the contents were expelled into 1 mL of 40 mM K_2_HPO_4_, 15 mM KH_2_PO_4_. Serial dilutions were plated onto minimal medium supplemented with 15 mM glucose as the carbon source. The number of colony-forming units was determined after 36 h of incubation at 30 °C. In all the cases, the data were corrected with the number of cells that swam into the buffer-containing capillaries.

### Growth experiments

*P. atrosepticum* SCRI1043 was cultured overnight in phosphate-free M9-based medium (PFM9) [93] supplemented with 20 mM glucose as the carbon source and 500 µM inorganic phosphate as the phosphate source. Cultures were washed and then diluted to an OD_600_ of 0.02 in PFM9 supplemented with different PacP ligands as the sole carbon or phosphorous source at a final concentration of 5 mM. Subsequently, 200 µL aliquots of these cultures were transferred into microwell plates, which were subsequently grown at 30 °C on a Bioscreen microbiological growth analyzer (Oy Growth Curves Ab Ltd., Helsinki, Finland) for 48 h.

### Bioinformatic analyses

Molecular docking was performed on PacP and phosphorylated compounds by DiffDock with 10 inference steps [94]. Homologs of the PacP (WP_011094075.1) N-terminal fragment (residues 7–197) were collected from RefSeq by BLAST (*E* value < 0.05) [95]. The sequences were aligned via MAFFT [96] and reduced to 98% redundancy using CD-HIT [97]. The sCache_2 domain regions from the aligned sequences (corresponding to residues 27–178 in PacP) were used for phylogenetic analyses [98]. A maximum likelihood tree was constructed using MEGA with the JTT model and 100 bootstraps [99]. The protein targets were selected on the basis of their location on the phylogenetic tree and the presence of key residues for ligand binding.

## Supplementary Information

Additional file 1: Supplementary figures. Fig. S1 Composition of the compound arrays PM1, PM2A, PM3B, and PM4A used for ligand screening. Fig. S2 Quantitative capillary chemotaxis assays of different *P. atrosepticum* SCRI1043 strains to 1 mM glycerol 3-phosphate (A) and 3-phosphoglycerate (B). Fig. S3 Changes in the midpoint of the protein unfolding transition (Tm) derived from thermal shift assays of domains R2, R3, and R4 with compound arrays PM1, PM2A, PM3B, and PM4A (see Fig. S1 for composition). Fig. S4 The importance of the binding site motif in ligand recognition. Microcalorimetric titration of a PacP-LBD mutant in which the amino acids of the binding site motif (Y86, H100, R105, Y121, K148, and Y167) have been replaced with alanine residues, with glycerol 3-phosphate and glyceraldehyde 3-phosphate.

Additional file 2: Supplementary tables. Table S1 Information on the phylogenetics, lifestyle, and isolation sources of strains containing chemoreceptors selected for study. Table S2 Strains, plasmids, and oligonucleotides used in this study. Table S3 Sequences of the proteins analyzed in this study. Table S4 Buffers used for protein purification and analysis.

Additional file 3: Members of the sCache_PC3 domain family.

## Data Availability

The source code and datasets used and/or analyzed during the current study are available at the GitHub (https://github.com/Jiawei-Xing/sCache_PC3) [100] and Zenodo (https://zenodo.org/records/15849485) [101] repositories under an MIT license. The following sequencing data were used in this study: WP_011094075.1 (PacP, ECA_RS12390): [60], RefSeq assembly: GCF_000011605.1. WP_136157342.1: https://www.ncbi.nlm.nih.gov/datasets/genome/GCF_003115845.1/, RefSeq assembly: GCF_003115845.1. WP_006464688.1: [102], RefSeq assembly: GCF_000300975.2. WP_158281851.1: https://www.ncbi.nlm.nih.gov/datasets/genome/GCF_003201855.1/, RefSeq assembly: GCF_003201855.1. WP_028444678.1: https://www.ncbi.nlm.nih.gov/datasets/genome/GCF_000428465.1/, RefSeq assembly: GCF_000428465.1. WP_174062248.1: https://www.ncbi.nlm.nih.gov/datasets/genome/GCF_013322225.1/, RefSeq assembly: GCF_013322225.1. WP_019915375.1: [69], RefSeq assembly: GCF_000527135.1. WP_028536266.1: https://www.ncbi.nlm.nih.gov/datasets/genome/GCF_000422925.1/, RefSeq assembly: GCF_000422925.1. WP_189529961.1: https://www.ncbi.nlm.nih.gov/datasets/genome/GCF_014652495.1/, RefSeq assembly: GCF_014652495.1. WP_028866120.1: https://www.ncbi.nlm.nih.gov/datasets/genome/GCF_000428725.1/, RefSeq assembly: GCF_000428725.1
