# Supplementary material for: Chemoreceptor family in plant-associated bacteria responds preferentially to the plant signal molecule glycerol 3-phosphate
Source: Genome Biol. 2025 Aug 29;26:260. doi: 10.1186/s13059-025-03703-6 (PMC12395807; doi:10.1186/s13059-025-03703-6)
Supplement: Supplementary file 1 — Additional file 1: Supplementary figures. Fig. S1 Composition of the compound arrays PM1, PM2A, PM3B, and PM4A used for ligand screening. Fig. S2 Quantitative capillary chemotaxis assays of different P. atrosepticum SCRI1043 strains to 1 mM glycerol 3-phosphate (A) and 3-phosphoglycerate (B). Fig. S3 Changes in the midpoint of the protein unfolding transition (Tm) derived from thermal shift assays of domains R2, R3, and R4 with compound arrays PM1, PM2A, PM3B, and PM4A (see Fig. S1 for composition). Fig. S4 The importance of the binding site motif in ligand recognition. Microcalorimetric titration of a PacP-LBD mutant in which the amino acids of the binding site motif (Y86, H100, R105, Y121, K148, and Y167) have been replaced with alanine residues, with glycerol 3-phosphate and glyceraldehyde 3-phosphate. [file 13059_2025_3703_MOESM1_ESM.docx]

**Additional file 1: Supplementary Figures**

**To**

**Chemoreceptor family in plant-associated bacteria responds preferentially to the plant signal molecule glycerol 3-phosphate**

**By**

Félix Velando^1#^, Jiawei Xing^2,3#§^, Roberta Genova^1#^, Jean Paul Cerna-Vargas^1,4^, Raquel Vázquez-Santiago^1^, Miguel A. Matilla^1^, Igor B. Zhulin^2,3*^, Tino Krell^1*^

^1^Department of Environmental Protection, Estación Experimental del Zaidín, Consejo Superior de Investigaciones Científicas, Granada, Spain

^2^Department of Microbiology, The Ohio State University, Columbus, USA

^3^Translational Data Analytics Institute, The Ohio State University, Columbus, USA

^4^Centro de Biotecnología y Genómica de Plantas CBGP, Universidad Politécnica de Madrid-Instituto Nacional de Investigación y Tecnología Agraria y Alimentaria/CSIC, Parque Científico y Tecnológico de la UPM, Pozuelo de Alarcón, Madrid, Spain

**Fig. S1) Composition of the compound arrays PM1, PM2A, PM3B and PM4A used for ligand screening.**

**
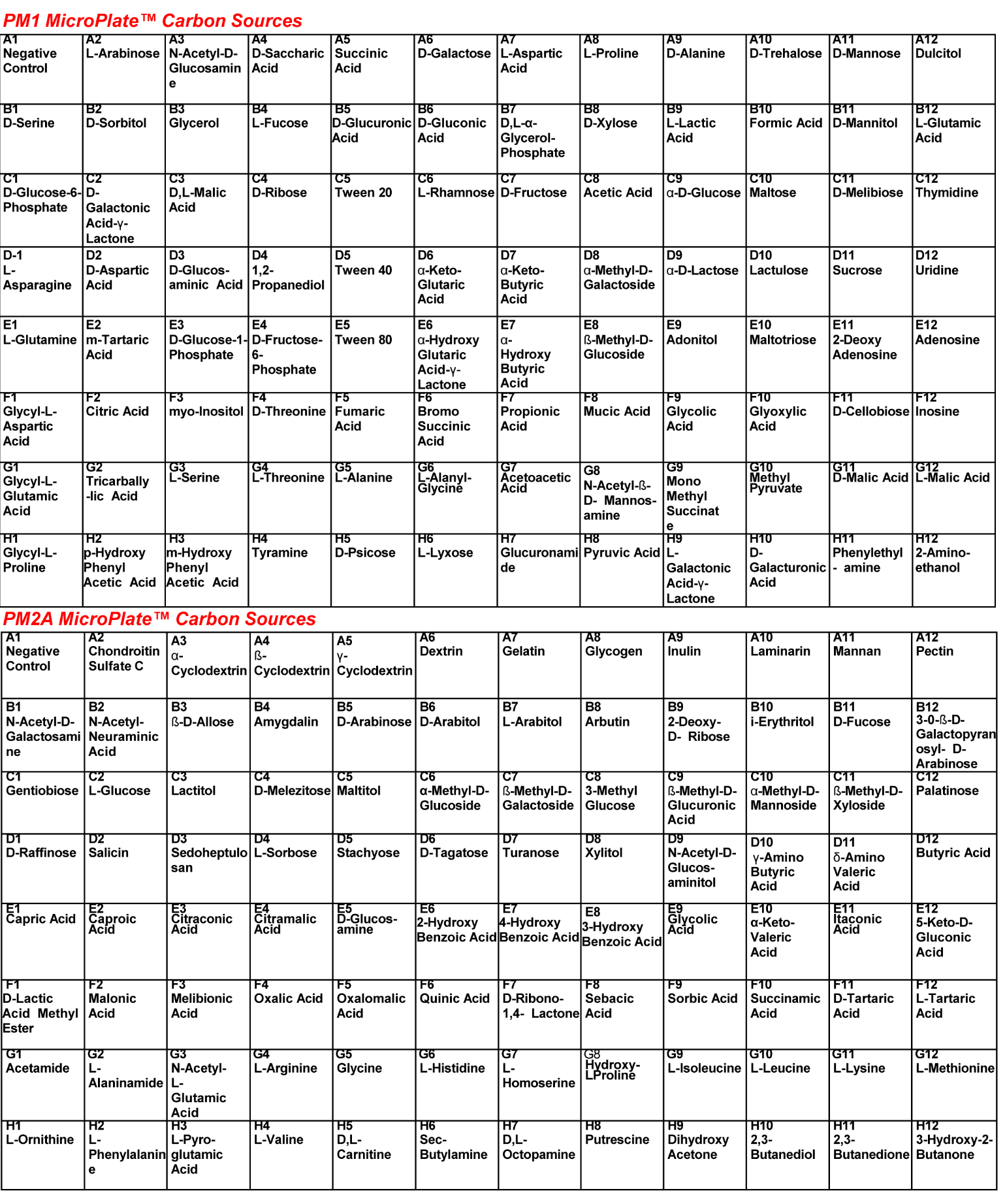
**

**
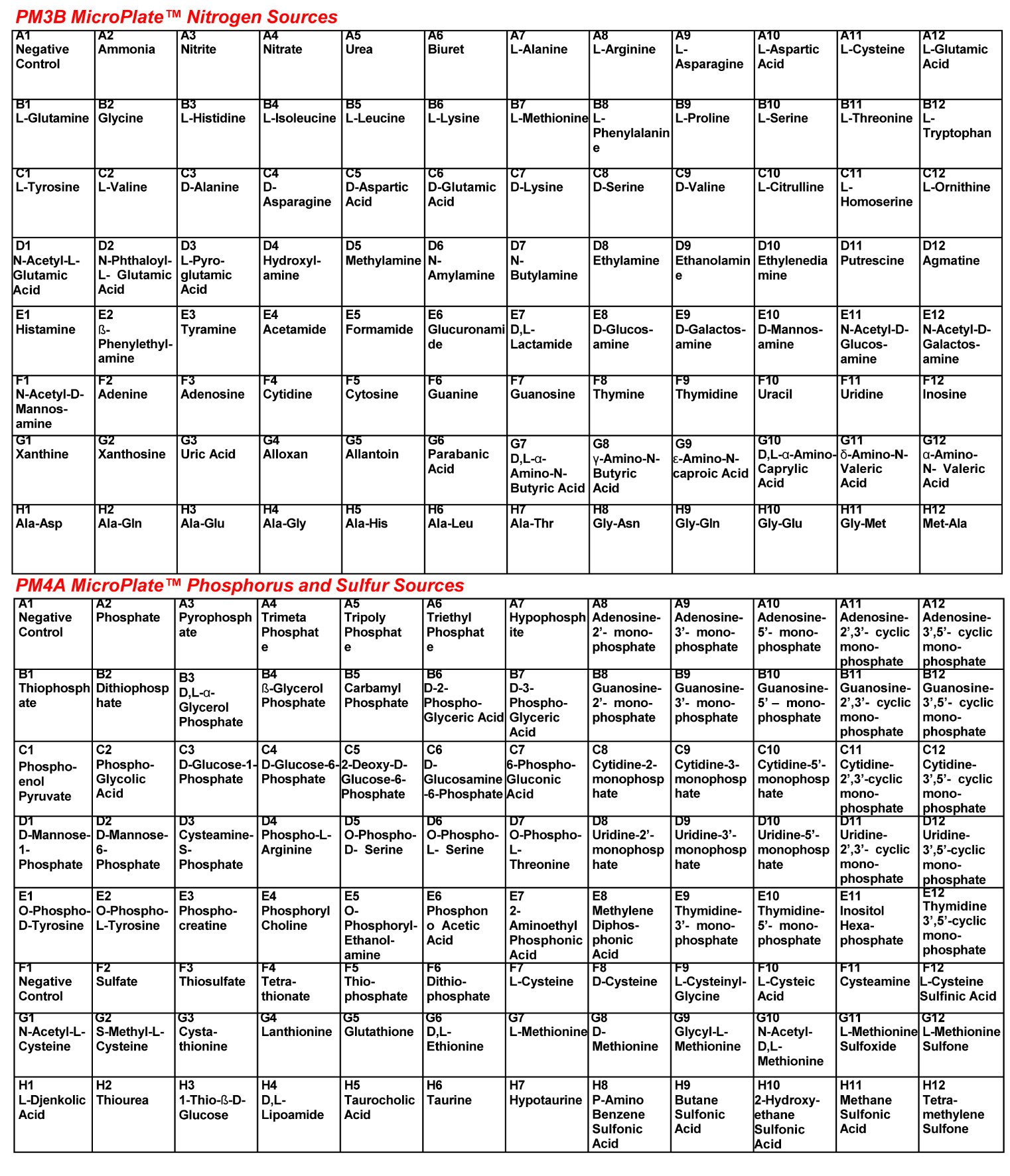
**

**Fig. S2) Quantitative capillary chemotaxis assays of different *P. atrosepticum* SCRI1043 strains to 1 mM Glycerol 3-phosphate (A) and 3-phosphoglycerate (B).** Wt: *P. atrosepticum* SCRI1043, Δ*cheA*: mutant deficient in cheA gene encoding the autokinase; WT + pBBR1MCS2_START: P. atrosepticum SCRI1043 containing the empty expression plasmid; M*pacP* + pBBR1MCS2_START: mutant in the *pacP* chemoreceptor gene (ECA_RS12390) containing the empty expression plasmid; M*pacP +* pBBR-ECA_RS12390*:* mutant in the *pacP* chemoreceptor gene (ECA_RS12390) containing a plasmid encoding the *pacP* gene (ECA_RS12390). Data are the means and standard deviations from at least three biological replicates conducted in triplicate. Data have been corrected with the number of cells that swam into buffer-containing capillaries, namely WT: 933 ± 753, ∆*cheA*: 895 ± 447, WT + pBBR1MCS2_START: 2013 ± 525, M*pacP* + pBBR1MCS2_START: 2064 ± 672, M*pacP* + pBBR_ECA_RS12390: 1209 ± 216.

**
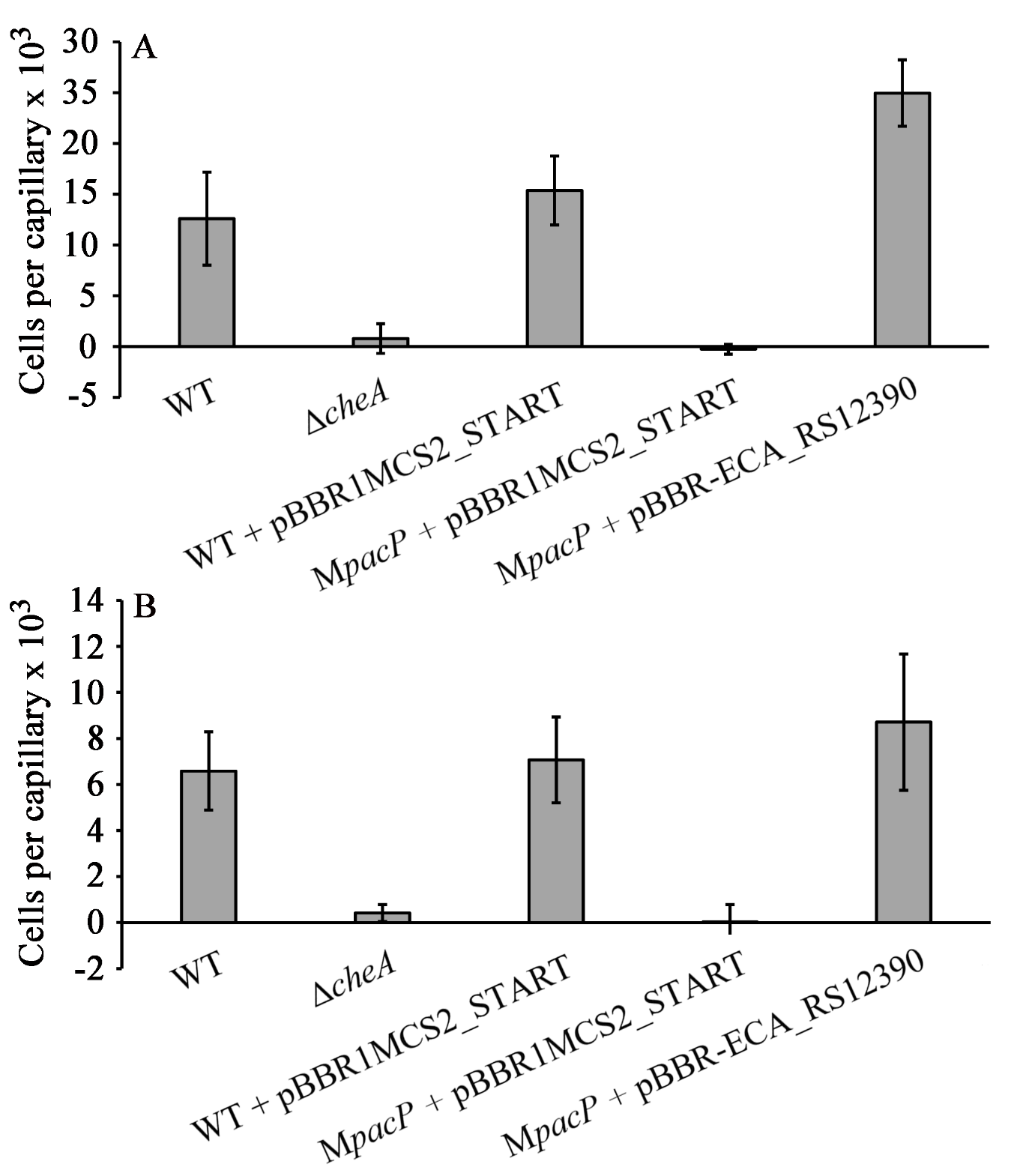
**

**Fig. S3) Changes in the midpoint of the protein unfolding transition (Tm) derived from thermal shift assays of domains R2, R3 and R4 with compound arrays PM1, PM2A, PM3B and PM4A (see Fig. S1 for composition).** Microcalorimetric titrations of R2 with maximal possible concentrations of formic acid, acetic acid, propionic acid, acetoacetic acid, caproic acid, D,L-aminocaprylic acid and L-cysteic acid did not reveal binding.

**
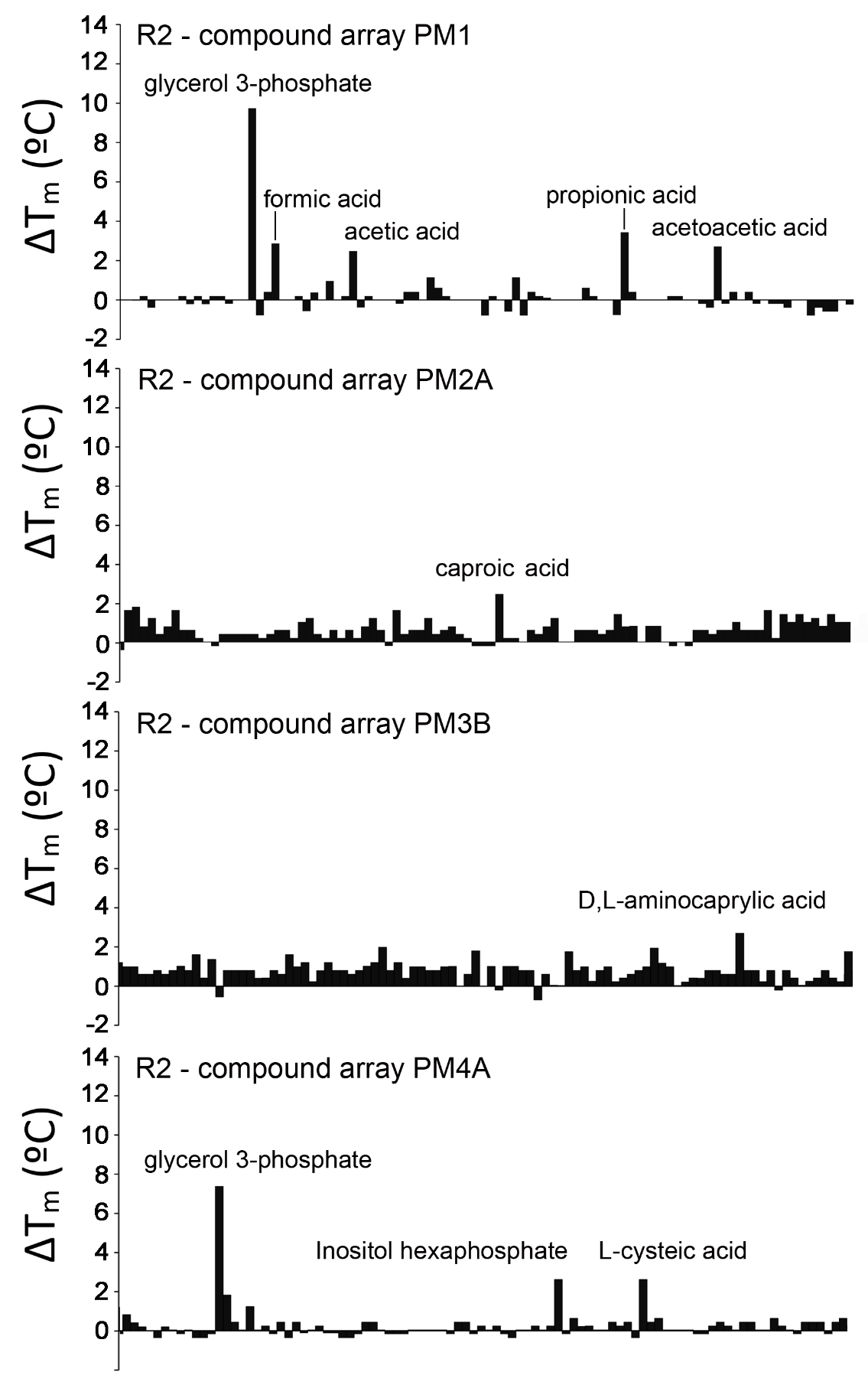
**

**

**

**

**

**Fig. S4) The importance of the binding site motif in ligand recognition.** Microcalorimetric titration of 36 μM of a PacP-LBD mutant in which the amino acids of the binding site motif (Y86, H100, R105, Y121, K148 and Y167) have been replaced with alanine residues, with 8 µl aliquots of 1 mM glycerol 3-phosphate and 2.5 mM glyceraldehyde 3-phosphate. The sequence of this protein is provided in Table S3. This protein was expressed from plasmid pET28b_ECA_RS12390mut (Table S2). Shown are the titration raw data. The scale of the y-axis corresponds to that of Fig. 1B showing the titration of PacP-LBD with glycerol 3-phosphate.

**

**
