# Supplementary material for: Chemoreceptor family in plant-associated bacteria responds preferentially to the plant signal molecule glycerol 3-phosphate
Source: Genome Biol. 2025 Aug 29;26:260. doi: 10.1186/s13059-025-03703-6 (PMC12395807; doi:10.1186/s13059-025-03703-6)
Supplement: Supplementary file 2 — Additional file 2: Supplementary tables. Table S1 Information on the phylogenetics, lifestyle, and isolation sources of strains containing chemoreceptors selected for study. Table S2 Strains, plasmids, and oligonucleotides used in this study. Table S3 Sequences of the proteins analyzed in this study. Table S4 Buffers used for protein purification and analysis. [file 13059_2025_3703_MOESM2_ESM.docx]

**Additional file 2: Supplementary Tables**

**To**

**Chemoreceptor family in plant-associated bacteria responds preferentially to the plant signal molecule glycerol 3-phosphate**

**By**

Félix Velando^1#^, Jiawei Xing^2,3#§^, Roberta Genova^1#^, Jean Paul Cerna-Vargas^1,4^, Raquel Vázquez-Santiago^1^, Miguel A. Matilla^1^, Igor B. Zhulin^2,3*^, Tino Krell^1*^

^1^Department of Environmental Protection, Estación Experimental del Zaidín, Consejo Superior de Investigaciones Científicas, Granada, Spain

^2^Department of Microbiology, The Ohio State University, Columbus, USA

^3^Translational Data Analytics Institute, The Ohio State University, Columbus, USA

^4^Centro de Biotecnología y Genómica de Plantas CBGP, Universidad Politécnica de Madrid-Instituto Nacional de Investigación y Tecnología Agraria y Alimentaria/CSIC, Parque Científico y Tecnológico de la UPM, Pozuelo de Alarcón, Madrid, Spain

**Table S1: Information on the phylogenetics, lifestyle and isolation sources of strains containing chemoreceptors selected for study.**

| **Rec.** | **Accession code** | **Phylogenetic lineage^a^** | **Isolation source** | **Lifestyle** | **References** |
| --- | --- | --- | --- | --- | --- |
| R1 | WP_011094075.1  (PacP, ECA_RS12390) | *Pectobacterium atrosepticum*  *Pectobacterium*  *Pectobacteriaceae*  Enterobacterales  Gammaproteobacteria | Stem of *Solanum tuberosum* (potato) | Plant pathogen that causes soft rot and blackleg disease in a number of crops, particularly potato, facultative anaerobic, secretes cell wall degrading enzymes. | [1,2] |
| R2 | WP_136157342.1 | *Brenneria roseae*  *Brenneria*  *Pectobacteriaceae*  Enterobacterales  Gammaproteobacteria | Infects oak trees, responsible for acute oak decline | Plant pathogen, facultative anaerobic, species of the genus *Brenneria* cause cankers, wilts and necrosis in a number of different trees. | [3,4] |
| R3 | WP_006464688.1 | *Herbaspirillum frisingense*  *Herbaspirillum*  *Oxalobacteraceae*  Burkholderiales  Betaproteobacteria | Leaves of *Miscanthus sacchariflorus* (Amur silvergrass) | Plant-associated, plant endophyte of gramineous plants. | [5], https://img.jgi.doe.gov/cgi-bin/m/main.cgi?section=TaxonDetail&page=taxonDetail&taxon_oid=2540341032 |
| R4 | WP_158281851.1 | *Rivicola pingtungensis*  *Rivihabitans*  *Aquaspirillaceae*  Neisseriales  Betaproteobacteria | Freshwater river | Able to perform denitrification. | [6,7] |
| R5 | WP_028444678.1 | *Chitinimonas koreensis*  *Chitinimonas*  *Chitinibacteraceae*  Neisseriales  Betaproteobacteria | Greenhouse soil cultivated with cucumber |  | [8] https://img.jgi.doe.gov/cgi-bin/m/main.cgi?section=TaxonDetail&page=taxonDetail&taxon_oid=2524614556 |
| R6 | WP_174062248.1 | *Agrobacterium larrymoorei*  *Agrobacterium*  *Rhizobium/Agrobacterium group*  *Rhizobiaceae*  Hyphomicrobiales  Alphaproteobacteria | Tumor on weeping fig tree *Ficus benjamina* | Plant pathogen, produces tumors | [9] https://img.jgi.doe.gov/cgi-bin/m/main.cgi?section=TaxonDetail&page=taxonDetail&taxon_oid=2540341200 |
| R7 | WP_019915375.1 | *Methyloversatilis discipulorum*  *Methyloversatilis*  *Sterolibacteriaceae*  Nitrosomonadales  Betaproteobacteria | Lake Washington | Methylotrophic | [10] |
| R8 | WP_028536266.1 | *Paludibacterium yongneupense*  *Paludibacterium*  *Chromobacteriaceae*  Neisseriales  Betaproteobacteria | Wetland soil | Nitrate reducer | [11] https://img.jgi.doe.gov/cgi-bin/m/main.cgi?section=TaxonDetail&page=taxonDetail&taxon_oid=2524023162 |
| R9 | WP_189529961.1 | *Paludibacterium paludis*  *Paludibacterium*  *Chromobacteriaceae*  Neisseriales  Betaproteobacteria | Marshland |  | [12] |
| R10 | WP_028866120.1 | *Psychromonas aquimarina*  *Psychromonas*  *Psychromonadaceae*  Alteromonadales  Gammaproteobacteria | Sediment adjacent to sperm whale carcasses | Psychrotrophic | [13] |

^a^ According to the NCBI Taxonomy browser [14].

**Table S2: Strains, plasmids and oligonucleotides used in this study.**

|  | **Genotype or relevant characteristics** | | **Ref.** |
| --- | --- | --- | --- |
| **Strains** | | | |
| *Escherichia coli* DH5α | F^–^ *endA1* *glnV44* *thi-1*  *recA1*  *relA1*  *gyrA96 deoR* *nupG* *purB20* φ80d*lacZ*ΔM15 Δ(*lacZYA-argF*)U169, hsdR17(*r_K_*^–^*m_K_*^+^), λ^–^ | | [15] |
| *E. coli* BL21(DE3) | F^–^ *ompT* *gal* *dcm* *lon* *hsdS_B_*(*r_B_*^–^*m_B_*^–^) λ(DE3 [*lacI* *lacUV5*-*T7p07* *ind1* *sam7* *nin5*]) [*malB*^+^]_K-12_(λ^S^) | | [16] |
| *E. coli* CC118λpir | *araD* Δ(*ara, leu*) Δ*lacZ74 phoA20 galK thi-1 rspE rpoB argE recA1* λ*pir* | | [17] |
| *E. coli* β2163 | (F^−^) RP4-2-Tc::Mu Δ*dapA*::(*erm-pir*); Km^R^ Em^R^ | | [18] |
| *Pectobacterium atrosepticum* SCRI1043 | Wild type strain | | [1] |
| *P. atrosepticum* SCRI1043 Δ*cheA* | SCRI1043 deletion mutant of *cheA*; KmR | | [19] |
| M*pacP* | Sm^R^, *ECA_RS12390*::pKNG101 | | This study |
| **Plasmids** | | | |
| pET28b(+) | Km^R^; protein expression plasmid | | Novagen |
| pET28b_ECA_RS12390 | Km^R^; pET28b(+) derivative encoding the LBD of the ECA_RS12390 (PacP) chemoreceptor | | This study^a^ |
| pET28b_ECA_RS12390mut | Km^R^; pET28b_ECA_RS12390 derivative encoding the LBD of the ECA_RS12390 (PacP) chemoreceptor in which amino acids Y86, H100, R105, Y121, K148 and Y167 have been replaced by alanine residues. | | This study^a^ |
| pET28b_WP_136157342.1 | Km^R^; pET28b(+) derivative encoding the LBD from the PWC17178.1 chemoreceptor from *Brenneria roseae* | | This study^a^ |
| pET28b_WP_006464688.1 | Km^R^; pET28b(+) derivative encoding the LBD of WP_006464688.1 from *Herbaspirillum frisingense* | | This study^a^ |
| pET28b_WP_158281851.1 | Km^R^; pET28b(+) derivative encoding the LBD of WP_158281851.1 from *Rivicola pingtungensis* | | This study^a^ |
| pET28b_WP_028444678.1 | Km^R^; pET28b(+) derivative encoding the LBD of WP_028444678.1 from *Chitinimonas koreensis* | | This study^a^ |
| pET28b_WP_174062248.1 | Km^R^; pET28b(+) derivative encoding the LBD of WP_174062248.1 from *Agrobacterium larrymoorei* | | This study^a^ |
| pET28b_WP_019915375.1 | Km^R^; pET28b(+) derivative encoding the LBD of WP_019915375.1 from *Methyloversatilis discipulorum* | | This study^a^ |
| pET28b_WP_028536266.1 | Km^R^; pET28b(+) derivative encoding the LBD of WP_028536266.1 from *Paludibacterium yongneupense* | | This study^a^ |
| pET28b_WP_189529961.1 | Km^R^; pET28b(+) derivative encoding the LBD of WP_189529961.1 from *Paludibacterium paludis* | | This study^a^ |
| pET28b_WP_028866120.1 | Km^R^; pET28b(+) derivative encoding the LBD of WP_028866120.1 from *Psychromonas aquimarina* | | This study^a^ |
| pGEM^®^-T | Ap^R^; PCR cloning vector | | Invitrogen |
| pGEM::ECA_RS12390 | Ap^R^; pGEM^®^-T derivative containing a 0.5-Kb fragment of *ECA_RS12390* | | This study |
| pKNG101 | Sm^R^; *oriR6K mob* | | [19] |
| pKNG101::ECA_RS12390 | Sm^R^; pKNG101 derivative containing a 0.5-Kb fragment of *ECA_RS12390* cloned and inserted into XbaI | | This study |
| pBBR1MCS2_START | Km^R^; *oriRK2 mobRK2* | | [20] |
| pBBR-ECA_RS12390 | Gm^R^; a 1.6-kb PCR fragment containing the *ECA_RS12390* (*pacP*) gene cloned into the NdeI/BamHI sites of pBBR1MCS2_START | | This study |
| **Oligonucleotides** | | | |
| **Name** | **Sequence (5’-3’)** | **Purpose** | |
| Fw ECA_RS12390 | AACCCGGGACACTCTATAACACGATGATG | Generation of pKNG101::ECA_RS12390 | |
| Rv ECA_RS12390 | TTCCCGGGCATTGTGCCAGAACGTCT |  |  |
| Fw ECA_RS12390_com | TAATCATATGTGAAATTACGAACCAGAATTGCA | Generation of pBBR-ECA_RS12390 | |
| Rv ECA_RS12390_com | TAATGGATCCTTATCTTGCACCCAGCGCCA |  |  |

^a^Constructed by GenScript.

**Table S3: Sequences of the proteins analyzed in this study.** The C-terminal extension containing the His-tag is shaded in yellow.

| **Protein name** | **Sequence** |
| --- | --- |
| ECA_RS17860-LBD  (PacP-LBD, R1) | MGSSHHHHHHSSGLVPRGSHMNTLYNTMMSERTGQLSTLVELAHSAAQKAYELEKSGQLSRDEAEKEAKRTIGSFHQGDRYFFVRGYTNDVNYVHPNPKRIGIIDANGGKEAGERYRASLQGNTIGTVIAEGTRPGQQNKVEKLYAVIKFEPWDWTIGYGDYIDDIQQTFWHNAL |
| PacP-LBDmut | MGSSHHHHHHSSGLVPRGSHMNTLYNTMMSERTGQLSTLVELAHSAAQKAYELEKSGQLSRDEAEKEAKRTIGSFHQGDR**A**FFVRGYTNDVNYVAPNPK**A**IGIIDANGGKEAGERARASLQGNTIGTVIAEGTRPGQQNKVEALYAVIKFEPWDWTIGYGDAIDDIQQTFWHNAL |
| WP_136157342-LBD (R2) | MGSSHHHHHHSSGLVPRGSHTLYKSMMHERTSQLSTLVELAHAAAQKSYDLEKSGQLSREEAENEAKRAIGSFHLNDRYFFVRGFTNDVNYVHPNPKRVGIVDAKGGKEAGERYRAALQDNTIGTVIARGTRPGTTDEVEKLYAIVKFEPWDWIIGYGDYIDDIQQAFWRD |
| WP_006464688-LBD (R3) | MGSSHHHHHHSSGLVPRGSHTLRRTMMEEREAQLSLLVTLAKAAAEKAQAQEQAGKLTREQAQAQAKMVIGSFAKEQNYYFVRGYSDDFNYVHPNPKRVGIQDKTAKEDGDRYRAALQGKEIGLLIAEGTRPNTTEKVQKLYAVTRFAPWDWTIGFGAYIDDINQAFYRN |
| WP_158281851-LBD (R4) | MGSSHHHHHHSSGLVPRGSHRANMISERQSQFGALVSQGKASLAYFHKLEADGKLSREEAQKLAKQAIASLHDGDRYLWMRDNSNDVNLVHPNPKRVNHADPDAKKKGDEYRQAMQGVEVGFLFSQGTRPGVEGNVGKLYALSLFQPWNWIIGFGGYLDDIEQVFWQRAFTM |
| WP_028444678-LBD (R5) | MGSSHHHHHHSSGLVPRGSHQLRQTMMEERRGQITMLLGLAEGMLKQYQALETAGKLSREEAQARATQALGALRSEDNYFFARNGDNVMLIHPKTERIGKVDLGSKVPDGRYSTAVYAEALQQSNPALVVIQTERPKSKDKVLLPKLNGVLKFEPWQWTVGIGFFIDDIDT |
| WP_174062248-LBD (R6) | MGSSHHHHHHSSGLVPRGSHQIKSSVNAIYEERYGMLRTQVQSSISILQSFYDKEKAGTLSREDAQKQAFAIVSSMKYVPDGYMFGYDYDVNMMFHPDPKRVGQNFKGKADSQGFAYRDELVRLARSGGGQVNFLGPKPGEQGDSFLKSSYAMAFEPWQIVVVTGVYVDDLQAQVRST |
| WP_019915375-LBD (R7) | MGSSHHHHHHSSGLVPRGSHEIRSTMMAEREARIVTLLKLSTGILQRYHEQEKAGTLTREQAQTHAREALLGLQSDDHYMFARSADDVLLAHVRKEKLGQKDNGGVAPDGRTNTDVYREALAKADPAFVTVPAKKPGGSEALPKMNGVTHFAPWAWTLGTGFFVDDIEQAFKS |
| WP_028536266-LBD (R8) | MGSSHHHHHHSSGLVPRGSHNLRQTLLQERRSQIAKIVHLAANQIGYYQLQEKRGVLTGAQAQKQALQVVRGLRYQGDYIFVRRFDGFPLVHGNPDKEGRPDPSGGGRQADGRSLVQTYRDGLAHQPFALVEVQTPRPGSERALPKISGVMKIEGWNWIVGFGVFVDDVDEAFRVQ |
| WP_189529961-LBD (R9) | MGSSHHHHHHSSGLVPRGSHDIRLTLIHEKQHAIRTQLTMANTVLEHYATAERQGQMTREQAQSAARSALAMLRDGDTYIFARDRDQVMVVHPKTDLIGKKGNGGMMPDGKTSVVEAYEAALGQGRIGFVEVPSTRPGSTESVQKLNGVMRFDAWNWTVGSGVYLHDITETFWQ |
| WP_028866120-LBD (R10) | MGSSHHHHHHSSGLVPRGSHNDGLLAAKSKQTQNMVDAAHAVMNGYHKQFKNGELSEDDAKALALNAVKTMRYDNGNYFWVNDYSPTIIMHPVKPQLAGRDLSGVKDTNGKHLYLEFVRVAKESGSGHVDYLWEKPGADDPLEKISYVKAFKPWGWIIGTGIYVDDVRDSTWQLAAD |

**Table S4: Buffers used for protein purification and analysis.** No expression in *E. coli* was observed for R8.

| **Protein** | **Purification Buffer** | | **Analysis buffer** |
| --- | --- | --- | --- |
|  | **Buffer A** | **Buffer B** |  |
| WP_011095117.1  (PacP-LBD, R1) | 20 mM Tris/HCl, 500 mM NaCl, 5 % (v/v) glycerol, 10 mM imidazole, 0.1 mM EDTA, pH 8.5 | 20 mM Tris/HCl, 500 mM NaCl, 5 % (v/v) glycerol, 500 mM imidazole, 0.1 mM EDTA, pH 8.5 | 20 mM HEPES, 150 mM NaCl, 10 % (v/v) glycerol, pH 8.5 |
| PacP-LBDmut | 20 mM Tris/HCl, 500 mM NaCl, 5 % (v/v) glycerol, 10 mM imidazole, 0.1 mM EDTA, pH 8.5 | 20 mM Tris/HCl, 500 mM NaCl, 5 % (v/v) glycerol, 500 mM imidazole, 0.1 mM EDTA, pH 8.5 | 20 mM HEPES, 150 mM NaCl, 10 % (v/v) glycerol, pH 8.5 |
| WP_136157342.1 (R2) | 20 mM Tris/HCl, 500 mM NaCl, 5 % (v/v) glycerol, 10 mM imidazole, 0.1 mM EDTA, pH 8.5 | 20 mM Tris/HCl, 500 mM NaCl, 5 % (v/v) glycerol, 500 mM imidazole, 0.1 mM EDTA, pH 8.5 | 5 mM MES, 5 mM PIPES, 5 mM Tris/HCl, 10 % (v/v) glycerol, pH 8.5 |
| WP_006464688.1  (R3) | 20 mM Tris/HCl, 500 mM NaCl, 5 % (v/v) glycerol, 10 mM imidazole, 0.1 mM EDTA, pH 6.5 | 20 mM Tris/HCl, 500 mM NaCl, 5 % (v/v) glycerol, 500 mM imidazole, 0.1 mM EDTA, pH 6.5 | 40 mM K_2_HPO_4_, 40 mM KH_2_PO_4_, 5 % (v/v) glycerol, pH 6.3 |
| WP_158281851.1  (R4) | 20 mM Tris/HCl, 500 mM NaCl, 5 % (v/v) glycerol, 10 mM imidazole, 0.1 mM EDTA, pH 6.5 | 20 mM Tris/HCl, 500 mM NaCl, 5 % (v/v) glycerol, 500 mM imidazole, 0.1 mM EDTA, pH 6.5 | 40 mM K_2_HPO_4_, 40 mM KH_2_PO_4_, 5 % (v/v) glycerol, pH 6.3 |
| WP_028444678.1  (R5) | 20 mM Tris/HCl, 500 mM NaCl, 5 % (v/v) glycerol, 10 mM imidazole, 0.1 mM EDTA, pH 8.5 | 20 mM Tris/HCl, 500 mM NaCl, 5 % (v/v) glycerol, 500 mM imidazole, 0.1 mM EDTA, pH 8.5 | 5 mM MES, 5 mM PIPES, 5 mM Tris/HCl, 150 mM NaCl, 1 mM EDTA, 10 % (v/v) glycerol, pH 7.4 |
| WP_174062248.1  (R6) | 20 mM Tris/HCl, 500 mM NaCl, 5 % (v/v) glycerol, 10 mM imidazole, 0.1 mM EDTA, pH 6.5 | 20 mM Tris/HCl, 500 mM NaCl, 5 % (v/v) glycerol, 500 mM imidazole, 0.1 mM EDTA, pH 6.5 | 100 mM K_2_HPO_4_, 100 mM KH_2_PO_4_, 200 mM NaCl, 1 mM EDTA, 5 % (v/v) glycerol, pH 6.5 |
| WP_019915375.1  (R7) | 20 mM Tris/HCl, 500 mM NaCl, 5 % (v/v) glycerol, 10 mM imidazole, 0.1 mM EDTA, pH 8.5 | 20 mM Tris/HCl, 500 mM NaCl, 5 % (v/v) glycerol, 500 mM imidazole, 0.1 mM EDTA, pH 8.5 | 100 mM K_2_HPO_4_, 100 mM KH_2_PO_4_, 200 mM NaCl, 1 mM EDTA, 5 % (v/v) glycerol, pH 8.5 |
| WP_189529961.1  (R9) | 20 mM Tris/HCl, 500 mM NaCl, 5 % (v/v) glycerol, 10 mM imidazole, 0.1 mM EDTA, pH 8.0 | 20 mM Tris/HCl, 500 mM NaCl, 5 % (v/v) glycerol, 500 mM imidazole, 0.1 mM EDTA, pH 8.0 | 100 mM K_2_HPO_4_, 100 mM KH_2_PO_4_, 200 mM NaCl, 1 mM EDTA, 5 % (v/v) glycerol, pH 6.0 |
| WP_028866120.1  (R10) | 20 mM Tris/HCl, 500 mM NaCl, 5 % (v/v) glycerol, 10 mM imidazole, 0.1 mM EDTA, pH 8.0 | 20 mM Tris/HCl, 500 mM NaCl, 5 % (v/v) glycerol, 500 mM imidazole, 0.1 mM EDTA, pH 8.0 | 100 mM K_2_HPO_4_, 100 mM KH_2_PO_4_, 200 mM NaCl, 1 mM EDTA, 5 % (v/v) glycerol, pH 7.5 |

**References**

1. Bell KS, Sebaihia M, Pritchard L, Holden MT, Hyman LJ, Holeva MC, et al. Genome sequence of the enterobacterial phytopathogen Erwinia carotovora subsp. atroseptica and characterization of virulence factors. Proc Natl Acad Sci U S A. 2004;101:11105–10.

2. Toth IK. Microbe Profile: Pectobacterium atrosepticum: an enemy at the door. Microbiology (Reading). 2022;168.

3. Brady C, Hunter G, Kirk S, Arnold D, Denman S. Description of Brenneria roseae sp. nov. and two subspecies, Brenneria roseae subspecies roseae ssp. nov and Brenneria roseae subspecies americana ssp. nov. isolated from symptomatic oak. Syst Appl Microbiol. 2014;37:396–401.

4. Hauben, L., Swings, J. Genus: Brenneria. DJ Brenner, NR Krieg, JT Staley (Eds), Bergey’s Manual of Systematic Bacteriology, The Proteobacteria, Part B: The Gammaproteobacteria, vol 2, Springer, New York. 2005;628–33.

5. Rothballer M, Eckert B, Schmid M, Fekete A, Schloter M, Lehner A, et al. Endophytic root colonization of gramineous plants by Herbaspirillum frisingense. FEMS Microbiol Ecol. 2008;66:85–95.

6. Sheu S-Y, Chen J-C, Young C-C, Chen W-M. Rivicola pingtungensis gen. nov., sp. nov., a new member of the family Neisseriaceae isolated from a freshwater river. Int J Syst Evol Microbiol. 2014;64:2009–16.

7. Huang K, He Y, Wang W, Jiang R, Zhang Y, Li J, et al. Temporal differentiation in the adaptation of functional bacteria to low-temperature stress in partial denitrification and anammox system. Environ Res. 2024;244:117933.

8. Kim B-Y, Weon H-Y, Yoo S-H, Chen W-M, Kwon S-W, Go S-J, et al. Chitinimonas koreensis sp. nov., isolated from greenhouse soil in Korea. Int J Syst Evol Microbiol. 2006;56:1761–4.

9. Bouzar H, Jones JB. Agrobacterium larrymoorei sp. nov., a pathogen isolated from aerial tumours of Ficus benjamina. Int J Syst Evol Microbiol. 2001;51:1023–6.

10. Smalley NE, Taipale S, De Marco P, Doronina NV, Kyrpides N, Shapiro N, et al. Functional and genomic diversity of methylotrophic Rhodocyclaceae: description of Methyloversatilis discipulorum sp. nov. Int J Syst Evol Microbiol. 2015;65:2227–33.

11. Kwon S-W, Kim B-Y, Kim W-G, Yoo K-H, Yoo S-H, Son J-A, et al. Paludibacterium yongneupense gen. nov., sp. nov., isolated from a wetland, Yongneup, in Korea. Int J Syst Evol Microbiol. 2008;58:190–4.

12. Sheu S-Y, Chen Z-H, Young C-C, Chen W-M. Paludibacterium paludis sp. nov., isolated from a marsh. Int J Syst Evol Microbiol. 2014;64:2497–502.

13. Miyazaki M, Nogi Y, Fujiwara Y, Horikoshi K. Psychromonas japonica sp. nov., Psychromonas aquimarina sp. nov., Psychromonas macrocephali sp. nov. and Psychromonas ossibalaenae sp. nov., psychrotrophic bacteria isolated from sediment adjacent to sperm whale carcasses off Kagoshima, Japan. Int J Syst Evol Microbiol. 2008;58:1709–14.

14. Schoch CL, Ciufo S, Domrachev M, Hotton CL, Kannan S, Khovanskaya R, et al. NCBI Taxonomy: a comprehensive update on curation, resources and tools. Database : the journal of biological databases and curation. 2020/08/08 ed. 2020;2020.

15. Woodcock DM, Crowther PJ, Doherty J, Jefferson S, DeCruz E, Noyer-Weidner M, et al. Quantitative evaluation of Escherichia coli host strains for tolerance to cytosine methylation in plasmid and phage recombinants. Nucleic acids research. 1989;17:3469–78.

16. Jeong H, Barbe V, Lee CH, Vallenet D, Yu DS, Choi SH, et al. Genome sequences of Escherichia coli B strains REL606 and BL21(DE3). Journal of molecular biology. 2009;394:644–52.

17. Herrero M, de Lorenzo V, Timmis KN. Transposon vectors containing non-antibiotic resistance selection markers for cloning and stable chromosomal insertion of foreign genes in gram-negative bacteria. Journal of bacteriology. 1990;172:6557–67.

18. Demarre G, Guerout AM, Matsumoto-Mashimo C, Rowe-Magnus DA, Marliere P, Mazel D. A new family of mobilizable suicide plasmids based on broad host range R388 plasmid (IncW) and RP4 plasmid (IncPalpha) conjugative machineries and their cognate Escherichia coli host strains. Res Microbiol. 2005;156:245–55.

19. Kaniga K, Delor I, Cornelis GR. A wide-host-range suicide vector for improving reverse genetics in gram-negative bacteria: inactivation of the blaA gene of Yersinia enterocolitica. Gene. 1991;109:137–41.

20. Obranic S, Babic F, Maravic-Vlahovicek G. Improvement of pBBR1MCS plasmids, a very useful series of broad-host-range cloning vectors. Plasmid. 2013;70:263–7.
